# Dynamic Integration of Value Information into a Common Probability Currency as a Theory for Flexible Decision Making

**DOI:** 10.1371/journal.pcbi.1004402

**Published:** 2015-09-22

**Authors:** Vassilios Christopoulos, Paul R. Schrater

**Affiliations:** 1 Division of Biology and Biological Engineering, California Institute of Technology, Pasadena, California, United States of America; 2 Department of Psychology, University of Minnesota, Minneapolis, Minnesota, United States of America; 3 Department of Computer Science & Engineering, University of Minnesota, Minneapolis, Minnesota, United States of America; Oxford University, UNITED KINGDOM

## Abstract

Decisions involve two fundamental problems, selecting goals and generating actions to pursue those goals. While simple decisions involve choosing a goal and pursuing it, humans evolved to survive in hostile dynamic environments where goal availability and value can change with time and previous actions, entangling goal decisions with action selection. Recent studies suggest the brain generates concurrent action-plans for competing goals, using online information to bias the competition until a single goal is pursued. This creates a challenging problem of integrating information across diverse types, including both the dynamic value of the goal and the costs of action. We model the computations underlying dynamic decision-making with disparate value types, using the probability of getting the highest pay-off with the least effort as a common currency that supports goal competition. This framework predicts many aspects of decision behavior that have eluded a common explanation.

## Author Summary

Choosing between alternative options requires assigning and integrating values along a multitude of dimensions. For instance, when buying a car, different cars may vary for their price, quality, fuel economy and more. Solving this problem requires finding a common currency to allow integration of disparate value dimensions. In dynamic decisions, in which the environment changes continuously, this multi-dimensional integration must be updated over time. Despite many years of research, it is still unclear how the brain integrates value information and makes decisions in the presence of competing alternatives. In the current study, we propose a probabilistic theory that allows dynamically integrating value information into a common currency. It builds on successful models in motor control and decision-making. It is comprised of a series of control schemes with each of them attached to an individual goal, generating an optimal action-plan to achieve that goal starting from the current state. The key novelty is the relative desirability computation that integrates good- and action- values to a single dynamic variable that weighs the individual action-plans as a function of state and time. By dynamically integrating value information, our theory models many key results in movement decisions that have previously eluded a common explanation.

## Introduction

A soccer player moves the ball down the field, looking for an open teammate or a chance to score a goal. Abstractly, the soccer player faces a ubiquitous but challenging decision problem. He/she must select between many competing goals while acting, whose costs and benefits can change dynamically during ongoing actions. In this game scenario, the attacker has options to pass the ball to one of his/her teammates. An undefended player is preferred, but this opportunity will soon be lost if the ball is not quickly passed. If all teammates are marked by opposing players, other alternatives like holding the ball and delaying the decision may be better. Critically, the best option is not immediately evident before acting. To decide which strategy to follow at a given moment requires *dynamically integrating* value information from disparate sources. This information is diverse relating to both the dynamic value of the goal (i.e., relative reward of the goal, probability that reward is available for that goal) and the dynamic action cost (i.e., cost of actions to pursue that goal, precision required), creating a challenging problem in integrating information across these diverse types in real time. Despite intense research in decision neuroscience, dynamic value integration into a common currency remains poorly understood.

Previous explanations fall into two categories. The *goods-based* theory [1–7] proposes that all the decision factors associated with an option are integrated into a subjective economic value independently computed for each alternative. This view is consistent with evidence suggesting convergence of value information in the prefrontal cortex [3–5, 7]. Critically, action planning starts only after a decision is made. While this view is sufficient for decisions like buying or renting a house, modifications are needed for decisions while acting. Alternatively, an *action-based* theory proposes that options have associated action-plans. According to this theory, when the brain is faced with multiple potential goals, it generates concurrent action-plans that compete for selection and uses value information to bias this competition until a single option is selected [8–14]. This theory has been received apparent support from neurophysiological [8–10, 15–19] and behavioral [11, 12, 20–23] studies. Although the action-based theory explains competition, it leaves mysterious how action cost is integrated with good value (also referred as stimulus value in some decision-making studies [13]) that have different currencies and how goods-based decisions that do not involve action competition are made. To solve complex decision problems, the brain must dynamically integrate all the factors that influence the desirability of engaging in an action-plan directed towards a goal.

We propose a theory of dynamic value integration that subsumes both goods-based and action-based theories. We provide a simple, computationally feasible way to integrate online information about the cost of actions and the value of goods into an evolving assessment of the *desirability* of each goal. By integrating value information into a common currency, our approach models many key results in decision tasks with competing goals that have eluded a common explanation, including trajectory averaging in rapid reaching tasks with multiple potential goals, a common explanation for errors due to competition including the global-effect paradigm in express saccadic movements [24], and a unified explanation for the pattern of errors due to competition in sequential decisions [25].

## Materials and Methods

This section describes analytically the computational theory developed in this study to model decisions in tasks with competing goals. We used a reaching task as a paradigm. Full details of the architecture and stochastic optimal control methodology that underlies the control schemes of our theory is in S1 Text for reaching and S2 Text for saccade models.

### Action selection in reaching tasks with competing goals

Stochastic optimal control has proven a powerful tool at modeling goal-directed movements, such as reaching [26], grasping [27] and walking [28] (for review see [29]). It involves solving for a policy *π* that maps states into actions **u**_*t*_ = *π*(**x**_*t*_) by minimizing a cost function penalizing actions and deviations from a goal. Despite the growing popularity of optimal control models, most of them are limited to tasks with single goals, because policies are easily defined towards a single goal. On the other hand, it is unclear how to define policies in the presence of multiple goals, each of which may provide different reward and may require different effort. The core difficulty is to develop a single policy that selects actions that pursue many targets but ultimately arrives at only one.

One of the simplest solutions is to carefully construct a composite cost function that incorporates all targets. However, naive applications of this approach can produce quite poor results. For instance, an additive mixture of quadratic cost functions is a new cost function with a minimum that does not lie at any of the competing targets. The difficulty is that quadratic cost functions do not capture the winner-take-all implicit reward structure, since mixtures of quadratics reward best for terminal positions in between targets. Even when such a cost function can be constructed, it can be very difficult to solve the policy, since these types of decision problems are P-SPACE complete—a class of problems more intractable than NP-complete. Any dynamic change in targets configuration requires a full re-computation, which makes the approach difficult to implement as a real-time control strategy [30].

To preserve simplicity, we propose to decompose the problem into policy solutions for the individual targets. The overall solution should involve following the best policy at each moment, given incoming information. We can construct a simple cost function that has this property using indicator variables *ν*(**x**_*t*_). The indicator variables encode the policy that has the lowest future expected value from each state—in other words, it categorizes the state space into regions where following one of the policies to a goal *i* is the best option. In essence, a goal *i* “owns” these regions of the state space. We can write the cost function that describes this problem as a *ν*-weighted mixture of individual cost functions $J_{j}^{\prime }s$:

$$\begin{array}{ccc} J & = & \sum_{j=1}^{N}\nu_{j}\left(x_{t}\right)J_{j}\left(x_{t},\pi_{j}\right)\\ J & = & \sum_{j=1}^{N}\nu_{j}\left(x_{t}\right)(\underset{{}_{J_{j}\left(x_{t},\pi_{j}\right)}}{\underset{︸}{{\left(x_{T_{j}}-Sp_{j}\right)}^{T}Q_{T_{j}}(x_{T_{j}}-Sp_{j})+\sum_{t=1}^{T_{j}}\pi_{j}{\left(x_{t}\right)}^{T}R\pi_{j}\left(x_{t}\right)}}) \end{array}$$ (1)

where *N* is the total number of targets and *ν*_*j*_ is the indicator variable associated with the target *j*. The cost function *J*_*j*_(**x**_*t*_, *π*_*j*_) describes the individual goal for reaching the target *j* starting from the current state **x**_*t*_ and following the policy *π*_*j*_ for time instances *t* = [*t*_1_, ⋯, *t*_*T*_*j*__]. *T*_*j*_ is the time-to-contact that target *j* and *S* is a matrix that picks out the hand and target positions from the state vector. The first term of the cost *J*_*j*_ is the accuracy cost that penalizes actions that drive the end-point of the reaching trajectory away from the target position **p**_*j*_. The second term is the motor command cost that penalizes the effort required to reach the target. Both the accuracy cost and the motor command cost characterize the “action cost” *V*_*π*_*j*__(**x**_*t*_) for implementing the policy *π*_*j*_ at the state **x**_*t*_. Matrices *Q*_*T*_*j*__ and *R* define the precision- and the control- dependent costs, respectively (see S1 Text for more details).

When there is no uncertainty as to which policy to implement at a given time and state (e.g., actual target location is known), the *ν*-weighted cost function in Eq (1) is equivalent to the classical optimal control problem. The best policy is given by the minimization of the cost function in Eq (1) with *ν*_*j*_ = 1 for the actual target *j* and *ν*_*i* ≠ *j*_ = 0 for the rest of the non-targets. However, when there is more than one competing target in the field, there is uncertainty about which policy to follow at each time and state. In this case, the best policy is given by minimizing the expected cost function with expectation across the probability distribution of the indicator variable *ν*. This minimization can be approximated by the weighted average of the minimization of the expected individual cost functions, Eq (2).

$$\begin{array}{c} \pi_{mix}\left(x_{t}\right)=\sum_{j=1}^{N}{\left\langle \nu_{j}\left(x_{t}\right)\right\rangle }_{\nu }\;\;\arg\;\min_{\pi_{j}}J_{j}\left(x_{t},\pi_{j}\right)=\sum_{j=1}^{N}{\left\langle \nu_{j}\left(x_{t}\right)\right\rangle }_{\nu }\pi_{j}^{*}(x_{t}) \end{array}$$ (2)

where ⟨.⟩_*ν*_ is the expected value across the probability distribution of the indicator variable *ν*, and $\pi_{j}^{*}(x_{t})$ is the optimal policy to reach goal *j* starting from the current state **x**_*t*_. For notational simplicity, we omit the * sign from the policy *π*, and from now on *π*_*j*_(**x**_*t*_) will indicate the *optimal* policy to achieve the goal *j* at state **x**_*t*_.

### Computing policy desirability

The first problem is to compute the weighting factor ⟨*ν*_*j*_(**x**_*t*_)⟩_*ν*_, which determines the contribution of each individual policy *π*_*j*_(**x**_*t*_) to the weighted average *π*_*mix*_(**x**_*t*_). Let’s consider for now that all the alternative targets have the same good values and hence the behavior is determined solely by the action costs. Recall that *V*_*π*_*j*__(**x**_*t*_) represents the value function—i.e., cost that is expected to accumulate from the current state **x**_*t*_ to target *j* including the accuracy penalty at the end of the movement, under the policy *π*_*j*_(**x**_*t*_). This cost partially characterizes the probability of achieving at least *V*_*π*_*j*__(**x**_*t*_) starting from state **x**(*t*) at time *t* and adopting the policy *π*_*j*_(**x**_*t*_) to reach the target *j*, Eq (3):

$$\begin{array}{c} P(V_{\pi_{j}}\left(x_{t}\right)|\pi_{j}\left(x_{t}\right),x_{t},\Delta t)=\lambda e^{-\frac{1}{\lambda }V_{\pi_{j}\left(x_{t}\right)}} \end{array}$$ (3)

where *λ* is the free “inverse temperature” parameter (S3 Text). This assumption can be taken as is, or justified from the path integral approach in [31] and [32]. The probability that the value function of the policy *π*_*j*_ at the current state **x**_*t*_ is lower than the rest of the alternatives *P*(*V*_*π*_*j*__(**x**_*t*_) < *V*_*π*_*i* ≠ *j*__(**x**_*t*_)) can be approximated by the softmax-type equation in Eq (4), which gives an estimate of the probability of *ν*_*j*_ at **x**_*t*_:

$$\begin{array}{c} P\left(V_{\pi_{j}}\left(x_{t}\right)<V_{\pi_{i\neq j}}\left(x_{t}\right)\right)\approx P\left(\nu_{j}|x_{t}\right)=\frac{\lambda e^{-\frac{1}{\lambda }V_{\pi_{j}\left(x_{t}\right)}}}{\sum_{i=1}^{N}\lambda e^{-\frac{1}{\lambda }V_{\pi_{i}\left(x_{t}\right)}}} \end{array}$$ (4)

where *N* is the total number of targets (i.e., and total number of policies) that are available at the current state.

Given that all targets have the same good values, the probability *P*(*ν*_*j*_|**x**_*t*_) characterizes the “relative desirability” *rD*(*π_j_*(**x**_*t*_)) of the policy *π*_*j*_ to pursue the goal *j* at a given state **x**_*t*_. It reflects how desirable is to follow the policy *π*_*j*_ at that state with respect to the alternatives. Therefore, we can write that:

$$\begin{array}{c} rD(\pi_{j}\left(x_{t}\right))=P\left(V_{\pi_{j}}\left(x_{t}\right)<V_{\pi_{i\neq j}}\left(x_{t}\right)\right) \end{array}$$ (5)

However, in a natural environment the alternative goals are usually attached with different values that we should take into account before making a decision. We integrate the good values into the relative desirability by computing the probability that pursing the goal *j* will result in overall higher pay-off *r*_*j*_ than the alternatives, *P*(*r*_*j*_ > *r*_*i* ≠ *j*_):

$$\begin{array}{c} rD(\pi_{j}\left(x_{t}\right))=P\left(V_{\pi_{j}}\left(x_{t}\right)<V_{\pi_{i\neq j}}\left(x_{t}\right)\right)P\left(r_{j}>r_{i\neq j}\right) \end{array}$$ (6)

To integrate the goods-related component on the relative desirability, we consider two cases:

1. The reward magnitude is fixed and equal for all targets, but the receipt of reward is probabilistic. In this case, the probability that the value of the target *j* is higher than the rest of the alternatives is given by the reward probability of this target *P*(*target* = *j*|*x*_*t*_) = *p*_*j*_:
  $$\begin{array}{c} P\left(r_{j}>r_{i\neq j}\right)=p_{j} \end{array}$$ (7)
2. The target provides a reward with probability *p*_*j*_, but the reward magnitude is not fixed. Instead, we assume that it follows a distribution $r_{j}∼\left(1-p_{j}\right)\delta \left(r_{j}\right)+p_{j}N\left(\mu_{j},\sigma_{j}^{2}\right)$, where *δ*(*r*_*j*_) is the Delta dirac function, and *μ*_*j*_ and *σ*_*j*_ are the mean and the standard deviation of the reward attached to the target *j*. For simplicity reasons, we focus on the case with two potential targets, in which the goal is to achieve the highest pay-off after *N* trials. In this case, the goods-related component of the desirability function is $P({\bar{r}}_{1}>{\bar{r}}_{2})$, where ${\bar{r}}_{j}=\frac{1}{N}\sum_{k=1}^{N}r_{j}\left(k\right),j=1,2$ is the net reward attached to the target *j*—i.e., the average reward received from the target *j* across *N* trials.

To compute the probability $P({\bar{r}}_{1}>{\bar{r}}_{2})$, we need the probability distribution of $P({\bar{r}}_{j}),j=1,2$. Given *p*(*n*) = *Binomial*(*n*, *p*_*j*_, *N*) is the probability of receiving *n*-times reward after *N* trials, the probability distribution of ${\bar{r}}_{j}$ is:

$$\begin{array}{c} P\left({\bar{r}}_{j}\right)=\sum_{n=0}^{N}p\left(n\right)(\frac{1}{N}\sum_{k=1}^{n}r_{j}(i)) \end{array}$$ (8)

We can show that a mean based on *n* samples has a Normal distribution $N(\frac{n}{N}\mu_{j},\frac{\sigma_{j}^{2}}{n})$. Therefore, the distribution of ${\bar{r}}_{j}$ can be written as:

$$\begin{array}{c} P\left({\bar{r}}_{j}\right)=\sum_{n=0}^{N}p\left(n\right)N({\bar{r}}_{j};\frac{n}{N}\mu_{j},\frac{\sigma_{j}^{2}}{n}) \end{array}$$ (9)

For a large number of trials *N* > > 0, *p*(*n*) is concentrated around *n* = *p*_*j*_ *N* and ${\bar{r}}_{j}∼N(p_{j}\mu_{j},\frac{\sigma_{j}^{2}}{p_{j}N}),j=1,2$. To compute $P\left({\bar{r}}_{1}>{\bar{r}}_{2}\right)=P\left({\bar{r}}_{1}-{\bar{r}}_{2}>0\right)$, we define a new random variable, $Z={\bar{r}}_{1}-{\bar{r}}_{2}$, which has Normal distribution with mean *p*_1_ *μ*_1_ − *p*_2_ *μ*_2_ and variance $\frac{\sigma_{1}^{2}}{p_{1}N}+\frac{\sigma_{2}^{2}}{p_{2}N}$. We can show that $P\left({\bar{r}}_{1}>{\bar{r}}_{2}\right)=P\left(Z>0\right)$ is given as:

$$\begin{array}{c} P\left({\bar{r}}_{1}>{\bar{r}}_{2}\right)=\frac{1}{2}erfc(\frac{p_{2}\mu_{2}-p_{1}\mu_{1}}{\sqrt{2(\frac{\sigma_{1}^{2}}{p_{1}N}+\frac{\sigma_{2}^{2}}{p_{2}N})}}) \end{array}$$ (10)

where *erfc* is the complementary error function. Using that *erfc*(*x*) = 1 − *erf*(*x*), where *erf* is the error function, we can write that:

$$\begin{array}{c} P\left({\bar{r}}_{1}>{\bar{r}}_{2}\right)=\frac{1}{2}+\frac{1}{2}erf(\frac{p_{1}\mu_{1}-p_{2}\mu_{2}}{\sqrt{2(\frac{\sigma_{1}^{2}}{p_{1}N}+\frac{\sigma_{2}^{2}}{p_{2}N})}})=CumNorm(Z;p_{1}\mu_{1}-p_{2}\mu_{2},\frac{\sigma_{1}^{2}}{p_{1}N}+\frac{\sigma_{2}^{2}}{p_{2}N}) \end{array}$$ (11)

This result is consistent with the common practice of modeling choice probabilities as a softmax function between options. For example, the cumulative normal distribution can be approximated by the following logistic function [33]:

$$\begin{array}{c} P\left(r_{1}>r_{2}\right)=l({\bar{r}}_{1}-{\bar{r}}_{2};p_{2}\mu_{2}-p_{1}\mu_{1},\sqrt{\frac{S}{1.6}}) \end{array}$$ (12)

where $S=\frac{\sigma_{1}^{2}}{p_{1}N}+\frac{\sigma_{2}^{2}}{p_{2}N}$.

### Target probability encodes the order of policies in sequential movement tasks

In the preceding sections we developed a theory for the case that targets are presented simultaneously and the expected reward depends only on successfully reaching the target—i.e. reward availability is not state- and time- dependent. However, decisions are not limited only to this case but often involve goals with time-dependent values. In this section, we extend our approach to model visuomotor tasks with sequential goals, focusing on a pentagon copying task.

The theory precedes as before, with a set of control schemes that instantiate policies *π*_*j*_(**x**_*t*_)—where (*j* = 1, ⋯ 5)—that drive the hand from the current state to the vertex *j*. However, to draw the shape in a proper spatial order, we cannot use the same policy mixing as with simultaneously presented goals. Instead, we have to take into account the sequential constraints that induce a temporal order across the vertices. We can conceive the vertices as potential goals that provide the same amount of reward, but with different probabilities (i.e., similar to scenario 2 in the reaching task) with the exception that we design the target probability to be time- and state- dependent, so that it encodes the order of policies for copying the pentagon. The target probability *P*(*vertex* = *j*|**x**_*t*_) describes the probability that the vertex *j* is the current goal of the task at the state **x**_*t*_ after departing from the vertex *j* − 1, or in other words, it describes the probability that we copy the segment defined by the two successive vertices *j* − 1 and *j*.

We define an indicator function *e*_*j*_ that is 1 if we arrive at vertex *j* and 0 otherwise.

$$\begin{array}{c} P\left(vertex=j|x_{t}\right)=P\left(e_{j}=0,e_{j-1}=1|x_{t}\right)=P\left(e_{j}=0|x_{t}\right)p\left(e_{j}=1|x_{t}\right)= \end{array}$$ (13)

$$\begin{array}{c} =(1-P(\tau_{arrive}^{j}<t))P\left(\tau_{arrive}^{j-1}<t\right) \end{array}$$ (14)

where $\tau_{arrive}^{j}$ is the time to arrive at vertex *j* -i.e, time to complete drawing the segment defined by the vertices *j* − 1 and *j*.

**Fig 1:**
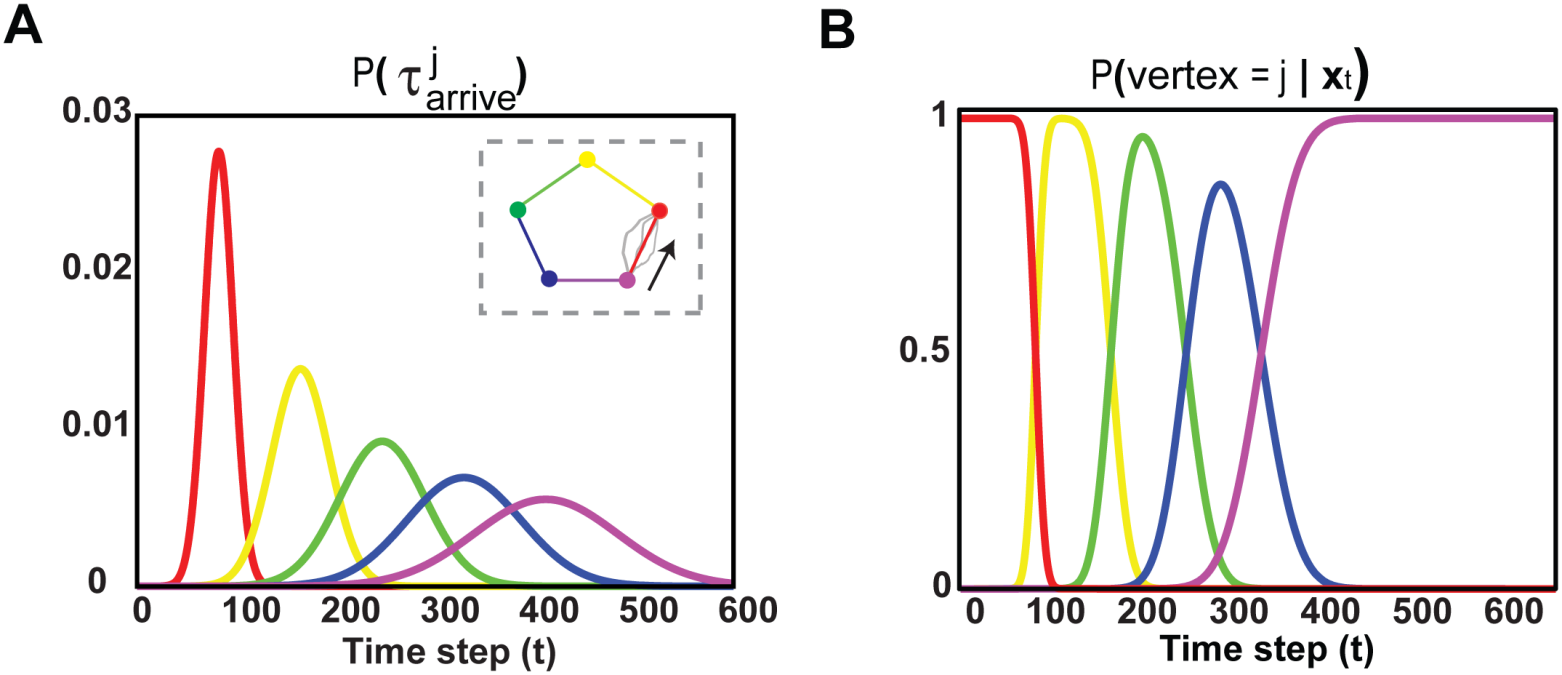
Encoding the order of policies in sequential movements. **A**: Probability distribution of time to arrive at vertex *j* starting from the original state at time *t* = 0 and visiting all the precedent vertices. Each color codes the segments and the vertices of the pentagon as shown in the right inset. The pentagon is copied counterclockwise (as indicated by the arrow) starting from the purple vertex at *t* = 0. The gray trajectories illustrate examples from the 100 reaches generated to estimate the probability distribution of time to arrive at vertex *k* given that we started from vertex *k* − 1, $P(\tau_{arrive}^{k}|\tau_{arrive}^{k-1})$. **B**: Probability distribution *P*(*vertex* = *j*|**x**_*t*_), which describes the probability to copy the segment defined by the two successive vertices *j* − 1 and *j* at state **x**_*t*_. This probability distribution is estimated at time *t* = 0 and when arriving at the next vertex, we condition on completion, and *P*(*vertex* = *j*|**x**_*t*_) is re-evaluated for the next vertices.

Let’s assume that we are copying the shape counterclockwise starting from the purple vertex (see right inset in Fig 1A), at the initial state at time *t* = 0. The probability distribution of time to arrive at vertex *j*, $\tau_{arrive}^{j}$, is given by Eq (15).

$$\begin{array}{c} P\left(\tau_{arrive}^{j}\right)=\sum_{k=1}^{j}P\left(\tau_{arrive}^{k}|\tau_{arrive}^{k-1}\right)P\left(\tau_{arrive}^{k-1}\right) \end{array}$$ (15)

where $P(\tau_{arrive}^{k}|\tau_{arrive}^{k-1})$ is the probability distribution of time to arrive at vertex *k* given that we started from vertex *k* − 1. We generated 100 trajectories between two successive vertices and found that $P(\tau_{arrive}^{k}|\tau_{arrive}^{k-1})$ can be approximated by a Normal distribution $N(\mu_{\tau_{arrive}},\sigma_{\tau_{arrive}}^{2})$. Using Eq (15), we show that $P(\tau_{arrive}^{j})$ is also Gaussian distribution, but with *j* times the mean and variance—$N(j\mu_{\tau_{arrive}},j\sigma_{\tau_{arrive}}^{2})$ as shown in Fig 1A. Considering that, we estimate that target probability *P*(*vertex* = *j*|**x**_*t*_), Fig 1B. Each time that we arrive at a vertex, we condition on completion, and *P*(*vertex* = *j*|**x**_*t*_) is re-evaluated for the next vertices.

## Results

### Model architecture

**Fig 2:**
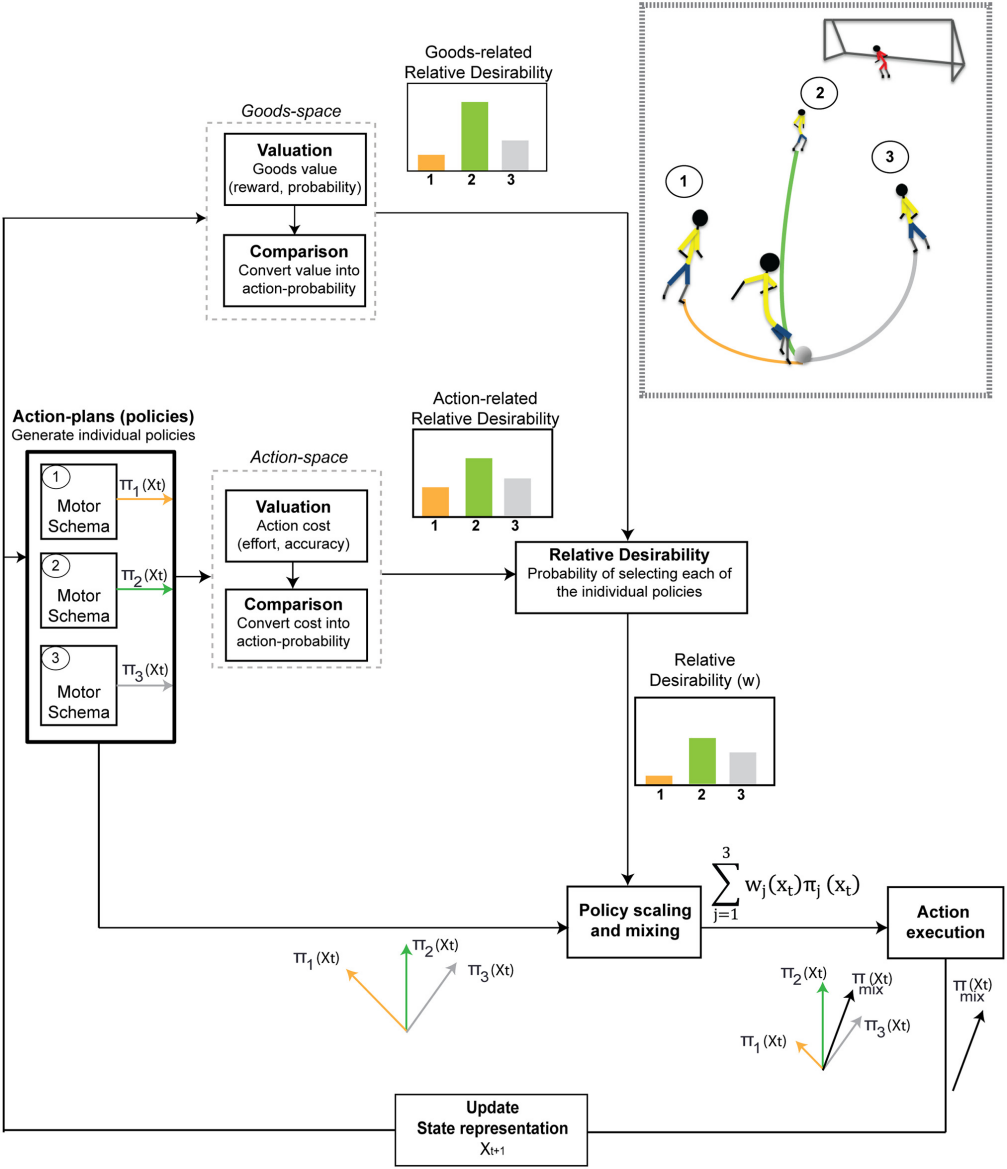
The architectural organization of the theory. It consists of multiple stochastic optimal control schemes where each of them is attached to a particular goal presented currently in the field. We illustrate the architecture of the theory using the hypothetical scenario of the soccer game, in which the player who is possessing the ball is presented with 3 alternative options—i.e., 3 teammates—located at different distances from the current state **x**_*t*_. In such a situation, the control schemes related to these options are triggered and generate 3 action plans (**u**_1_ = *π*_1_(**x**_*t*_), **u**_2_ = *π*_2_(**x**_*t*_) and **u**_3_ = *π*_3_(**x**_*t*_)) to pursue each of the individual options. At each time *t*, desirabilities of the each policy in terms of action cost and good value are computed separately, then combined into an overall desirability. The action cost of each policy is the cost-to-go of the remaining actions that would occur if the policy were followed from the current state **x**_*t*_ to the target. These action costs are converted into a relative desirability that characterizes the probability that implementing this policy will have the lowest cost relative to the alternative policies. Similarly, the good value attached to each policy is evaluated in the goods-space and is converted into a relative desirability that characterizes the probability that implementing that policy (i.e., select the goal *i*) will result in highest reward compare to the alternative options, from the current state **x**_*t*_. These two desirabilities are combined to give what we call “relative-desirability” value, which reflects the degree to which the individual policy *π*_*i*_ is desirable to follow, at the given time and state, with respect to the other available policies. The overall policy that the player follows is a time-varying weighted mixture of the individual policies using the desirability value as weighted factor. Because relative desirability is time- and state- dependent, the weighted mixture of policies produces a range of behavior from “winner-take-all” (i.e., pass the ball) to “spatial averaging” (i.e., keep the ball and delay your decision).

The basic architecture of the model is a set of control schemes, associated with individual goals, Fig 2. Each scheme is a stochastic optimal control system that generates both a goal-specific *policy* *π*_*j*_, which is a mapping between states and best-actions, and an action-cost function that computes the expected control costs to achieve the goal *j* from any state (see S1 Text for more details). It is important to note that a policy is not particular a sequence of actions—rather it is a controller that tells you what action-plan **u**_*j*_ (i.e., sequence of actions *u*_*i*_) to take from a state **x**_*t*_ to the goal (i.e., *π*_*j*_(**x**_*t*_) = **u**_*j*_ = [*u*_*t*_, *u*_*t*+1_, ⋯ *u*_*t*_*end*__]). In addition, the action-cost function is a map *cost*(*j*) = *V*_*π*_*j*__(**x**_*t*_) that gives the expected action cost from each state to the goal.

Let’s reconsider the soccer game scenario and assume a situation in which the player has 3 alternative options to pass the ball (i.e., 3 unmarked teammates) at different distances from the current state **x**_*t*_. In such a situation, the control schemes related to these options become active and suggest 3 action-plans (**u**_1_ = *π*_1_(**x**_*t*_), **u**_2_ = *π*_2_(**x**_*t*_) and **u**_3_ = *π*_3_(**x**_*t*_)) to pursue the individual options. Each of the alternative action-plans is assigned with value related to the option itself (e.g., teammates’ performance, distance of the teammates to the goalie) and with cost required to implement this plan (e.g., effort). For instance, it requires less effort to pass the ball to the nearby teammate No.1, but the distant teammate No.2 is considered a better option, because he/she is closer to the opponent goalie. While the game progresses, the cost of the action-plans and the estimates of the values of the alternative options change continuously. To make a correct choice, the player should integrate the incoming information online and while acting. However, the value of the options and the cost of the actions have different “currencies”, making the value integration a challenging procedure. The proposed theory uses a probabilistic approach to dynamically integrate value information from disparate sources into a common currency that we call the *relative desirability* function *w*(**x**_*t*_). While common currency usually refers to integration in value space, relative desirability combines in the space of policy weights. Using relative desirability, integration of disparate values is accomplished by combining each different type of value in its own space, then computing the relative impact of that value on the set of available policies.

### Relative desirability function

The crux of our approach is that to make a decision, we only need to know what is the current best option and whether we can achieve it. This changes the complex problem of converting action costs to good values into a simple problem of maximizing the chances of getting the best of the alternatives that are currently available. To integrate value information with different “currencies”, we compute the probability of achieving the most rewarding option from a given time and state. This probability has both action-related and goods-related components with an intuitive interpretation: the probability of getting the highest reward with the least effort. We call this value relative desirability (rD) because it quantifies the attractiveness of the policy *π* for each goal *i* from state **x**_*t*_ relative to the alternative options:

$$\begin{array}{c} rD(\pi_{i}\left(x_{t}\right))=P(cost\left(i\right)<cost\left(j\neq i\right)|x_{t})P(reward\left(i\right)>reward\left(j\neq i\right)|x_{t}) \end{array}$$ (16)

The first term is the “action-related” component of the relative desirability and describes the probability that pursuing the goal *i* has lowest cost relative to alternatives, at the given state **x**_*t*_. The second term refers to the “goods-related” component and describes the probability that selecting the goal *i* will result in highest reward compared to the alternatives, at the current state **x**_*t*_. Note that the relative desirability values of the alternative options are normalized so that they all sum to 1.

**Fig 3:**
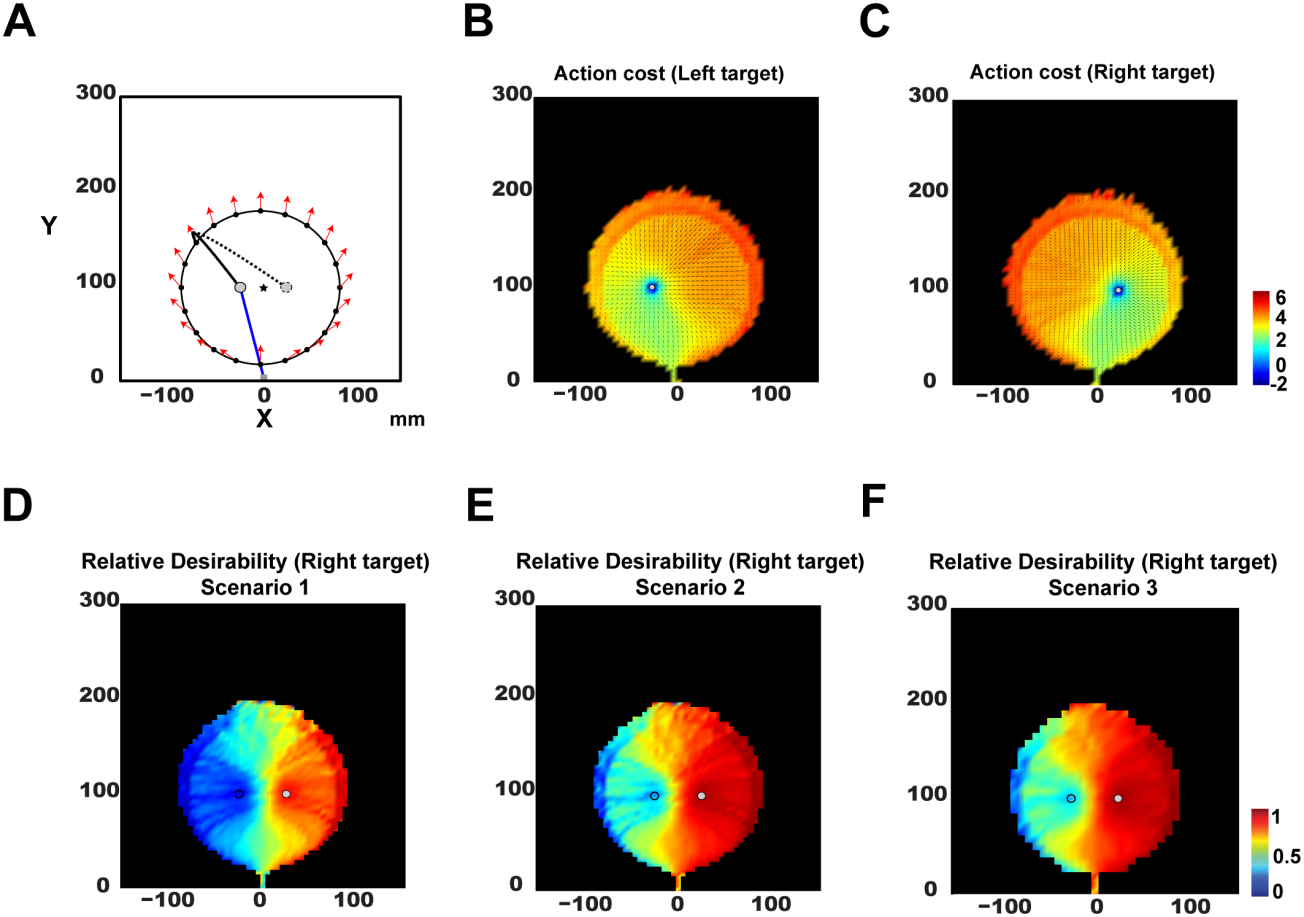
Relative desirability function in reaching movements with multiple potential targets. **A**: A method followed to visualize the relative desirability function of two competing reaching policies (see results section for more details). **B**: Heat map of the log-transformed action cost for reaching the left target (gray circle) starting from different states. Red and blue regions correspond to high and low cost states, respectively. The black arrows describe the average hand velocity at a given state. **C**: Similar to panel *B* but for the right target. **D**: Heat map of the relative desirability function at different states to reach to the right target, when both targets provide the same amount of reward with equal probability. **E**: Similar to *D*, but for a scenario in which the right target provides the same amount of reward with the left one, but with 4 times higher probability. **F**: Similar to *D*, but for a scenario in which the mean reward provided by right target is 4 times higher than then one provided by the left target.

To illustrate the relative desirability function, consider a reaching task with two potential targets presented in left (target *L*) and right (target *R*) visual fields (gray circles in Fig 3A). For any state **x**_*t*_ where the policy to the right target is more “desirable” than to the left target, we have the following inequality:

$$\begin{array}{c} rD(\pi_{R}\left(x_{t}\right))>rD(\pi_{L}\left(x_{t}\right)) \end{array}$$ (17)

This inequality predicts two extreme reaching behaviors—a direct movement to the target *R* (i.e., winner-take-all) when *rD*(*π_R_*(**x**_*t*_)) > > *rD* (*π_L_*(**x**_*t*_)), and a spatial averaging movement towards an intermediate position between the two targets when *rD*(*π_R_*(**x**_*t*_)) ≈ *rD*(*π_L_*(**x**_*t*_)). Rearranging this equation, using *P*(*reward*(*L*) > *reward*(*R*)) = 1 − *P*(*reward*(*R*) > *reward*(*L*)) we see that the relative desirability to pursue the target *R* increases with the odds that target *L* has more reward and lower cost:

$$\begin{array}{c} rD(\pi_{R}\left(x_{t}\right))>rD(\pi_{L}\left|x_{t}\right))\to (\frac{P(reward(R)>reward(L))}{1-P(reward(R)>reward(L))})(\frac{P(cost\left(R\right)<cost\left(L\right)|x_{t})}{1-P(cost\left(R\right)<cost\left(L\right)|x_{t})})>1 \end{array}$$ (18)

To gain more insight on how action cost and good value influence the reaching behavior, we visualize the relative desirability to reach the right target in 3 scenarios (the desirability related to the left target is a mirror image of the right one):

- **Scenario 1: Both targets provide the same reward magnitude with equal probability**.

For this case,

$$\begin{array}{c} P(reward(R)>reward(L))=1-P(reward(R)>reward(L)) \end{array}$$

which means target R is more desirable when

$$\begin{array}{c} P(cost\left(R\right)>cost\left(L\right)|x_{t})<0.5 \end{array}$$

Now the action cost (and hence relative desirability) is a function of the hand-state, making them difficult to illustrate. For a point-mass hand in 2D, the hand state is captured by the 4D position-velocity. To visualize this 4D relative desirability map in two dimensions, we “slice” through the 4D position-velocity space by making velocity a function of position in the following way. All trajectories are constrained to start at position (0, 0) with zero velocity. We then allow the trajectory to arrive at one of a set of spatial positions (100 total) around a circle of radius 85% of the distance between the start point and the midpoint (black star) of the two potential targets. For each of these points, we constrain the hand velocity to have direction (red arrows in Fig 3A) in line with the start point (gray square in Fig 3A) and the hand position on the circle (black dots in Fig 3A). We set the magnitude of the velocities to match the speed of the optimal reaching movement at 85% of completion (blue trace for left target in Fig 3A). From each position-velocity pair on the circle, we sample 100 optimal movements to each of the two targets (solid and discontinuous traces are illustrated examples for reaching the left and the right target, respectively). We discretize the space and compute the action cost to reach the targets from each state—the expected cost from each state to the goal following the policy for that goal, including an accuracy penalty at the end of the movement. Fig 3B and 3C depict these action costs, where blue indicates low cost and red indicates high cost, respectively. Fig 3D illustrates the action costs converted into relative desirability values to reach the right target (indicted by a solid gray circle), where blue and red regions correspond to states with low and high desirability, respectively. Notice desirability increases rapidly as the reach approaches a target, resulting in winner-take-all selection of an action-plan once moving definitely towards a target. However, when the hand position is about the same distance from both targets (greenish areas) there is no dominant policy, leading to strong competition and spatial averaging of the competing policies.

- **Scenario 2: Both targets provide the same reward magnitude but with different probabilities**.

In this case, desirability also depends on the probability of reward. Since both targets provide the same amount of reward, but with different probabilities, the goods-related term simplifies:

$$\begin{array}{c} P(reward(R)>reward(L))=P\left(target=R\right)=p_{R} \end{array}$$

where *p*_*R*_ describes the probability of earning reward by pursuing the right target. Hence, the target *R* is more desirable in a state **x**_*t*_ when

$$\begin{array}{c} P(cost\left(R\right)>cost\left(L\right)|x_{t})<p_{R} \end{array}$$

The relative desirability function for the right target is illustrated in Fig 3E, when *p*_*R*_ is 4 times higher than the probability of the left target (*p*_*R*_ = 0.8, *p*_*L*_ = 0.2). The right target is more desirable for most states (reddish areas), unless the hand position is already nearby the left target (blue areas), predicting frequent winner-take-all behavior -i.e., direct reaches to the right target.

- **Scenario 3: Probability and reward magnitude differ between the two targets**.

More generally, the reward magnitude attached to each target is not fixed, but both the reward magnitude and reward probability vary. We assume that target *j* provides a reward with probability *p*_*j*_, and that the magnitude follows a Normal distribution with mean *μ*_*j*_ and standard deviation *σ*_*j*_. Hence, the distribution of the rewards attached to the left target (L) and right target (R) is a mixture of distributions:

$$\begin{array}{c} reward\left(L\right)∼\left(1-p_{L}\right)\delta (reward(L))+p_{L}N\left(\mu_{L},\sigma_{L}^{2}\right) \end{array}$$ (19)

$$\begin{array}{c} reward\left(R\right)∼\left(1-p_{R}\right)\delta (reward(R))+p_{R}N\left(\mu_{R},\sigma_{R}^{2}\right) \end{array}$$ (20)

where *δ* is the Dirac function.

In visuomotor decision tasks, the ultimate goal is usually to achieve the highest reward after *N* trials. In this case, the probability that the right target provides overall higher reward than the left one over *N* trials can be approximated by a logistic function *l* with argument *p*_*R*_ *μ*_*R*_ − *p*_*L*_ *μ*_*L*_ (see Materials and Methods section for more details). When the reward values are precisely encoded, this simplifies to:

$$\begin{array}{c} P(reward(R)>reward(L))\approx l(p_{R}\mu_{R}-p_{L}\mu_{L}) \end{array}$$ (21)

Hence, pursuing the target *R* is more desirable in a state **x**_*t*_ when

$$\begin{array}{c} P(cost\left(R\right)>cost\left(L\right)|x_{t})<l(p_{R}\mu_{R}-p_{L}\mu_{L}) \end{array}$$ (22)

Fig 3F illustrates the heat map of the relative desirability values at different states of the policy to reach the right target (solid gray circle), when both targets have the same reward probability *p*_*L*_ = *p*_*R*_ = 0.5, but *μ*_*R*_ = 4*μ*_*L*_, i.e. *reward*(*R*) ∼ 0.5*δ*(*reward*(*R*)) + 0.5*N*(2, 1) and *reward*(*L*) ∼ 0.5*δ*(*reward*(*L*)) + 0.5*N*(0.5, 1). Similar to the previous scenario, reaching behavior is dominated mostly by the goods-related component and consequently reaching the right target is more desirable than reaching the left target for most states (reddish areas), leading frequently to “winner-take-all” behavior.

### Desirability predicts reaching behavior in decision tasks with multiple potential goals

**Fig 4:**
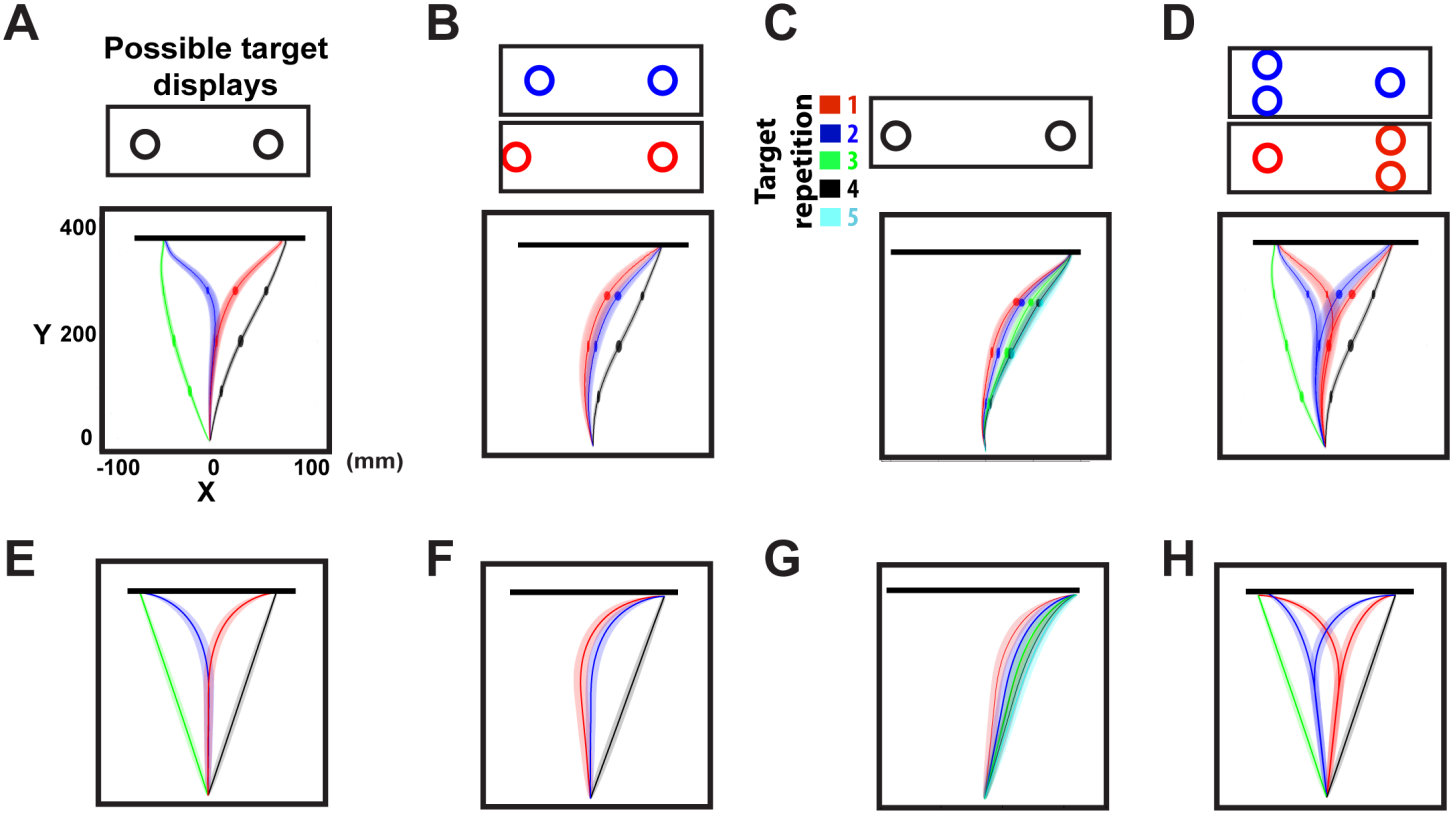
Rapid reaching movements in tasks with competing targets. Top row illustrates experimental results in rapid reaching tasks with multiple potential targets [12, 22, 23] (images are reproduced with permission of the authors). When the target position is known prior to movement onset, reaches are made directly to that target (black and green traces in **A**), otherwise, reaches aim to an intermediate location, before correcting in-flight to the cued target (red and blue traces in **A**). The competition between the two reaching policies that results in spatial averaging movements, is biased by the spatial distribution of the targets (**B**), by recent trial history (**C**) and the number of targets presented in each visual field (**D**). The bottom row (**E-H**) illustrates the simulated reaching movements generated in tasks with multiple potential targets. Each bottom panel corresponds to the reaching condition described on the top panels.

Several studies have shown that reaching decisions made while acting follow a “delay-and-mix” policy, with the mixing affected by target configuration and task properties [11, 12, 22, 23]. Subjects were trained to perform rapid reaching movements either to a single target or to two equidistant, equiprobable targets (i.e., actual target location is unknown prior to movement onset in two-target trials). Black and green traces in Fig 4A show single-target trials, characterized by trajectories straight to the target location. Red and blue traces show the delay-and-mix policy for reaches in two-target trials—an initial reaching movement towards an intermediate position between the two stimuli followed by corrective movements after the target was revealed. Relative desirability predicts this behavior (Fig 3D), for equiprobable reward (*scenario 1*). In this case, the relative desirability is determined solely by the distance from the current hand position to the targets. Since targets are equidistant, the reaching costs are comparable and hence the two competing policies have about the same desirability values for states between the origin and the target locations (see the greenish areas in Fig 3D). Hence, the weighted mixture of policies produces spatial averaging trajectories (red and blue traces in Fig 4E). Note that each controller *i*, which is associated with the potential target *i*, generates an optimal policy *π*_*i*_(**x**_*t*_) to reach that target starting from the current state **x**_*t*_. On single-target trials, the actual location of the target is known prior to movement onset and hence the desirability is 1 for the cued target. Consequently the simulated reaches are made directly to the actual target location (green and black traces in Fig 4E).

The competition between policies is also modulated by spatial location of the targets [12]. When one of the targets was shifted, reaching trajectories shifted towards a new intermediate position Fig 4B. This behavior is also captured by our framework—perturbing the spatial distribution of the potential targets, the weighted policy is also perturbed in the same direction Fig 4F. This finding is somehow counterintuitive, since the targets are no longer equidistant from the origin and it would be expected that the simulated reach responses would be biased towards the closer target. However, the magnitude of the perturbation is too small to change the action costs enough to significantly bias the competition. More significant are the action costs required to change direction once the target is revealed, and these costs are symmetric between targets.

Reaching behavior is also influenced by goods-related decision variables, like target probability. When subjects were informed that the potential targets were not equiprobable, the reach responses were biased towards the target with the highest reward probability [11]. This finding is consistent with relative desirability predictions in *scenario 2*—targets with higher reward probabilities are more desirable than the alternative options for most of the states. Reward probabilities learned via feedback can also be modeled in the same framework. Instead of informing subjects directly about target probabilities, the experimenters generated a block of trials in which one of the targets was consecutively cued for action [22]. Subjects showed a bias towards the cued target that accumulated across trials (Fig 4C) consistent with probability learning. We modeled this paradigm by updating the reward probability using a simple reinforcement learning algorithm (see S4 Text for more details). In line with the experimental findings, the simulated reach responses were increasingly biased to the target location that was consecutively cued for action on the past trials, Fig 4G.

Unlike most value computation methods, our approach can make strong predictions for what happens when additional targets are introduced. A previous study showed that by varying the number of potential targets, reaching movements were biased towards the side of space that contains more targets [12], Fig 4D. Our approach predicts this effect due to normalization across policies. When there are more targets in one hemifield than the other, there are more alternative reaching policies towards this space biasing the competition to that side, Fig 4H. Overall, these findings show that weighting individual policies with the relative desirability values can explain many aspects of human behavior in reaching decisions with competing goals.

### Desirability predicts errors in oculomotor decision tasks

**Fig 5:**
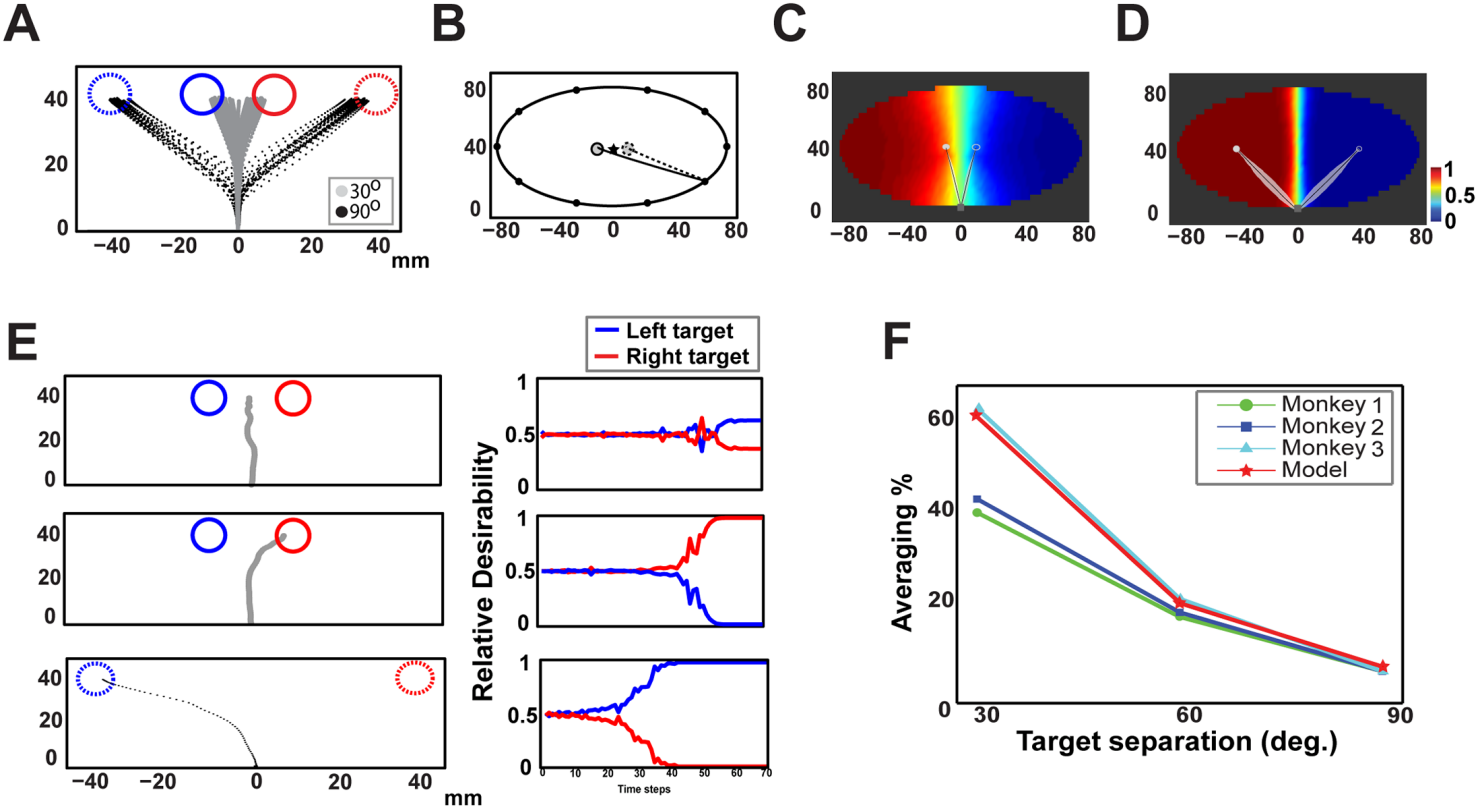
Saccadic movements in tasks with competing targets. **A**: Simulated saccadic movements for pair of targets with 30° (gray traces) and 90° (black traces) target separation. **B**: A method followed to visualize the relative desirability function of two competing saccadic policies (see results section for more details). **C**: Heat map of the relative desirability function at different states to saccade to the left target, at a 30° target separation. Red and blue regions corresponds to high and low desirability states, respectively. Black traces correspond to averaged trajectories in single-target trials. Notice the strong competition between the two saccadic policies (greenish areas). **D**: Similar to panel *C*, but for 90° target separation. In this case, targets are located in areas with no competition between the two policies (red and blue regions). **E**: Examples of saccadic movements (left column) with the corresponding time course of the relative desirability of the two policies (right column). The first two rows illustrate characteristic examples from 30° target separation, in which competition results primarily in saccade averaging (top panel) and less frequently in correct movements (middle panel). The bottom row shows a characteristic example from 90° target separation, in which the competition is resolved almost immediately after saccadic onset, producing almost no errors. **F**: Percentage of simulated averaging saccades for different degrees of target separation (red line)—green, blue and cyan lines describe the percentage of averaging saccades performed by 3 monkeys [24].

A good theory should predict not only successful decisions, but also decisions that result in errors in behavior. Experimental studies provide fairly clear evidence that humans and animals follow a “delay-and-mix” behavior even when it appears pathological. A typical example is the “global effect” paradigm that occurs frequently in oculomotor decisions with competing goals. When two equally rewarded targets are placed in close proximity—less than 30° angular distance—and the subject is free to choose between them, saccade trajectories usually end on intermediate locations between targets [24, 34, 35]. To test whether our theory can capture this phenomenon, we modeled the saccadic movements to individual targets using optimal control theory (see S2 Text for more details) and ran a series of simulated oculomotor decision tasks. Consistent with the experimental findings, the simulated eye movements land primarily in a position between the two targets for 30° target separation (gray traces in Fig 5A), whereas they aim directly to one of them for 90° target separation (black traces in Fig 5A).

We visualize the relative desirability of the left target (i.e., desirability to saccade to the left target) at different states, both for 30° and 90° target separation. We followed a similar procedure as for the reaching case but used an ellipse. Particularly, individual saccadic movements are constrained to start at (0, 0) and arrive at one of the sequence (100 total) of spatial positions with *zero velocity* around an ellipse with center intermediate between the two targets (black star), with minor axis twice the distance between the origin and the center of the ellipse, and major axis double the length of the minor axis (Fig 5B). For each position on the ellipse, we generate 100 optimal saccadic movements and evaluate the relative desirability to saccade to the left target (solid gray circle) at different states. Fig 5C depicts the heat-map of the relative desirability for 30° target separation. The black traces represent the average trajectories for direct saccadic movements, when only a single target is presented. Notice that regions defined by the starting position (0, 0) (gray square) and the locations of the targets is characterized by states with strong competition between the two saccadic policies (greenish areas). Consequently the weighted mixture of policies results frequently in spatial averaging movements that land between the two targets. On the other hand, when the targets are placed in distance, such as the 90° case presented in Fig 5D, the targets are located in areas in which one of the policies clearly dominates the other, and therefore the competition is easily resolved.

Fig 5E shows examples of saccadic movements (left column) with the corresponding time course of relative desirability values to saccade to the left and the right target (right column). The first two rows show trials from the 30° target separation task, where the competition between the two saccadic policies results in global effect (upper panels) and saccadic movement to the right target (middle panels). The two policies have about the same relative desirability values at different states resulting in a strong competition. Because saccades are ballistic with little opportunity for correction during the trajectory, competition produces the global effect paradigm. However, if the competition is resolved shortly after saccade onset, the trajectory ends up to one of the targets. On the other hand, when the two targets are placed in distance, the competition is easily resolved and the mixture of the policies generates direct movements to one of the targets (lower panel).

These findings suggest that the competition between alternative policies depends on the geometrical configuration of the targets. We quantified the effects of the targets’ spatial distribution to eye movements by computing the percentage of averaging saccades against the target separation. The results presented in Fig 5F (red line) indicate that averaging saccades were more frequent for 30° target separation and fell off gradually as the distance between the targets increases (see the Discussion section for more details on how competition leads to errors in behavior). This finding is also in line with experimental results from an oculomotor decision study with express saccadic movements in non-human primates (green, blue and cyan lines in Fig 5F describe the performance of 3 monkeys [24]).

### Desirability explains the competition in sequential decision tasks

In previous sections we considered decisions between multiple competing goals. However, ecological decisions are not limited only to simultaneous goals, but often involve choices between goals with time-dependent values. Time-dependent values mean that some of the goals may spoil or have limited period of worth such that they must be reached within a time window or temporal order. A characteristic example is sequential decision tasks that require a chain of decisions between successive goals. Substantial evidence suggests that the production of sequential movements involves concurrent representation of individual policies associated with the sequential goals that are internally activated before the order is imposed upon them [25, 36–39]. To model these tasks using our approach, the critical issue is how to mix the individual control policies. State-dependent policy mixing as described previously will dramatically fail, since the desirability values do not take into account the temporal constraints. However, it is relatively easy to incorporate the sequential constraints and time-dependence into the goods-related component of the relative desirability function. We illustrate how sequential decision tasks can be modeled using a simulated copying task used in neurophysiological [25, 40] and brain imaging studies [41, 42].

Copying geometrical shapes can be conceived as sequential decisions with goal-directed movements from one vertex (i.e., target) of the shape to another in a proper spatial order. To model this, each controller *j* provides a policy *π*_*j*_ to reach the vertex *j* starting from the current state. We encode the order of the policies using a time-dependent target reward probability *p*(*vertex* = *j*|**x**_*t*_) that describes the probability that *vertex* *j* is the current goal of the task at state **x**_*t*_ (see Materials and Methods section for more details). In fact, it describes the probability to copy the segment defined by the successive vertices *j* − 1 and *j* at a given state **x**_*t*_.

**Fig 6:**
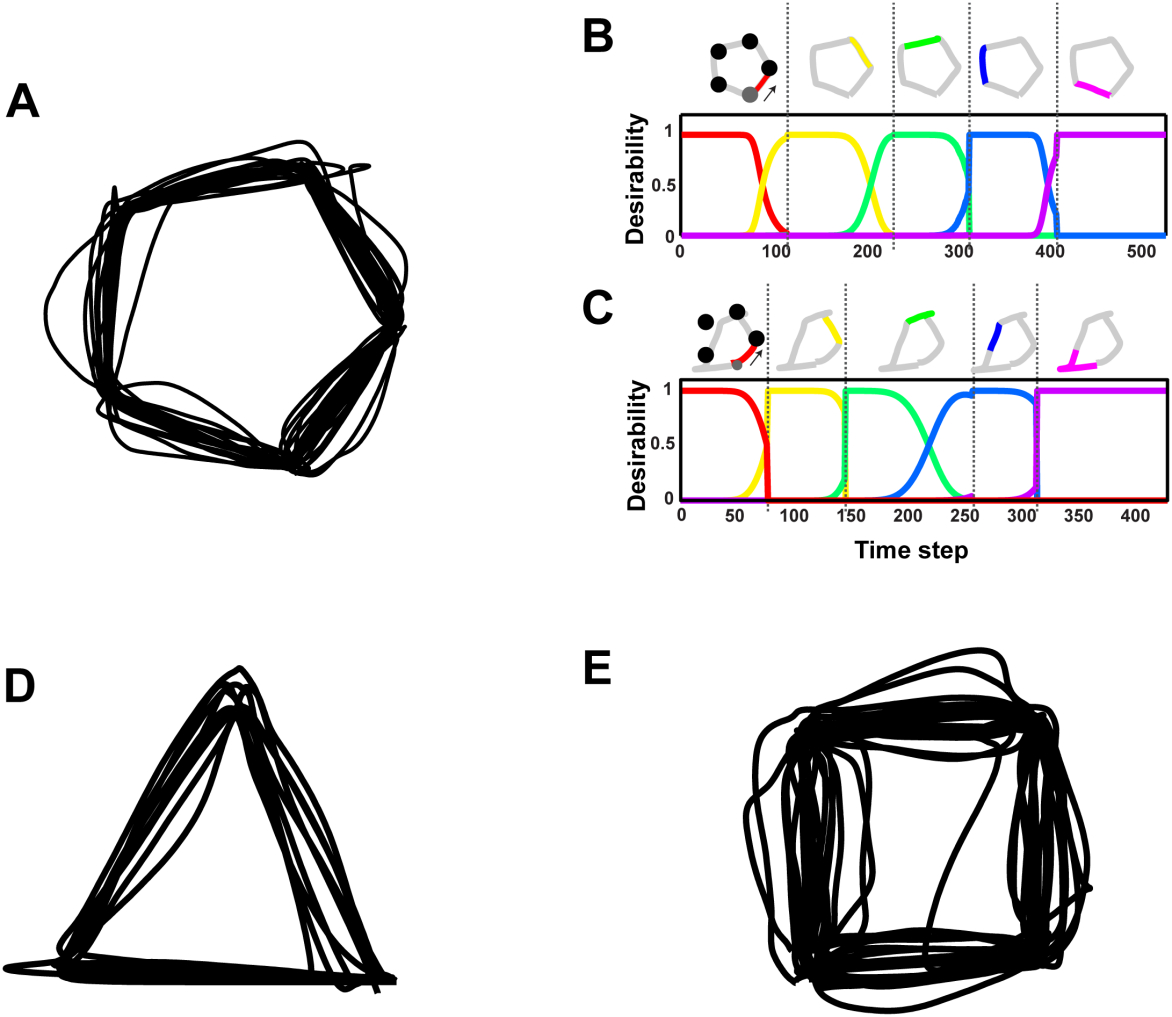
Sequential movements. **A**: Examples of simulated trajectories for continuously copying a pentagon. **B**: Time course of the relative desirability values of the 5 individual policies (i.e., 5 segments) in a successful trial for copying a pentagon. The line colors correspond to the segments of the pentagon as shown in the top panel. The shape was copied counterclockwise (as indicated by the arrow) starting from the gray vertex. Each of the horizontal discontinuous lines indicate the completion time of copying the current segment. Notice that the desirability of the current segment peaks immediately after the start of drawing that segment and falls down gradually, whereas the desirability of the following segment starts rising while copying the current segment. Because of that, the consecutive segments compete for action selection frequently producing error trials, as illustrated in panel **C**. Finally, the panels (**D**) and (**E**) depict examples of simulated trajectories for continuously copying an equilateral triangle and a square, respectively, counterclockwise starting from the bottom right vertex.

We evaluated the theory in a simulated copying task with 3 geometrical shapes (i.e., equilateral triangle, square and pentagon). Examples of movement trajectories from the pentagon task is shown in Fig 6A. Fig 6B depicts the time course of the relative desirability values of the segments from a successful trial. The desirability of each segment peaks once the model starts copying that segment and falls down gradually, whereas the desirability of the following segment starts rising while copying the current segment. Notice that the competition is stronger for middle segments than the first or the last segment in the sequence. Consequently, errors, such as rounding of corners and transposition errors (i.e., copying other segments than the current one in the sequence) are more frequent when copying the middle segments of the shape, than during the execution of the early or late segments. These simulation results are congruent with studies showing that human/animal accuracy in serial order tasks is better during early or late elements in the sequence [25, 43]. A characteristic example is illustrated in Fig 6C, in which the competition between copying the “blue” and the “green” segments resulted in an error trial.

Notice also that the temporal pattern of desirability values is congruent with populations of neural activity in prefrontal cortex during the copying task that encode each of the segments [25]. The strength of the neuronal population corresponding to a segment predicted the serial position of the segment in the motor sequence, providing a neural basis for Lashley’s hypothesis. Interestingly, the temporal evolution of the population activities resembles the temporal evolution of the relative desirabilities of policies in our theory. This finding provides a direct neural correlate of relative desirability suggesting that the computations in our model are biologically plausible. Finally, Fig 6D and 6E illustrate examples of movement trajectories for copying an equilateral triangle and a square.

## Discussion

How the brain dynamically selects between alternatives challenges a widely used model of decisions that posit comparisons of abstract representations of “goods” [1–7]. According to this model, the brain integrates all the decision variables of an option into a subjective economic value and makes a decision by comparing the values of the alternative options. Most importantly, the comparison is taking place within the space of goods, independent of the sensorimotor contingencies of choice [5]. While abstract representation of values have been found in brain areas like orbitrofrontal cortex (OFC) and ventromedial prefrontal cortex (vmPFC) [4, 44], these representations do not necessarily exclude the involvement of sensorimotor areas in decisions between actions. Recent studies provide evidence for an “action-based” theory involving competition between concurrent prepared actions associated with alternative goals [9, 10, 12–14, 17]. The main line of evidence of this theory is recent findings from neurophysiological studies [15, 16, 45, 46] and studies that involve reversible inactivation of sensorimotor regions [47, 48]. According to these studies, sensorimotor structures, such as the lateral intraparietal area (LIP) [48], the dorsal premotor cortex (dPM) [16], the superior colliculus (SC) [47] and the parietal reach region (PRR) [45, 46] are causally involved in decisions.

Despite the attractiveness of the “action-based” theory to model decisions between actions, what has been missing is a computational theory that can combine good values (e.g., money, juice reward) with action costs (e.g., amount of effort) into an integrated theory of dynamic decision-making. Previous studies have used principles from Statistical Decision Theory (SDT) to model human behavior in visuomotor decisions [49]. According to these studies, action-selection can be modeled as a decision problem that maximizes the desirableness of outcomes, where desirableness can be captured by an expected gain function. Despite the significant contribution of these studies to the understanding of the mechanisms of visuomotor decisions, they have focused mostly on static environments, in which the availability and the value of an option do not change with time and previous actions. Additionally, the expected gain functions usually involve the integration of decision values that have the same currency, such as expected monetary gains and losses—e.g., humans perform rapid reaching movements towards displays with regions that, if touched within a boundary lead to monetary reward, otherwise to monetary penalty [50, 51]. In the current study, we propose a probabilistic model that shows how value information from disparate sources with different “currencies” can be integrated in a manner that is both online and can be updated during action execution. The model is based on stochastic optimal control theory and is consistent with the view that decision and action are merged in a parallel rather than serial order. It is comprised of a series of control schemes that each of them is attached to an individual goal and generates a policy to achieve that goal starting from the current state. The key to our model is the relative desirability value that integrates the action costs and good values to a single variable that weighs the individual control policies as a function of state and time. It has intuitive meaning of the probability of getting the highest pay-off with the least cost following a specific policy at a given time and state. Because the desirability is state- and time- dependent, the weighted mixture of policies produces a range of behavior automatically, from “winner-take-all” to “weighted averaging”. By dynamically integrating in terms of probabilities across policies, relative-desirability varies with decision context. Relative desirability’s effective exchange rate changes whenever action costs increase or decrease, the set of options change, or the value of goods increase. Moreover, relative desirability is dynamic and state-dependent, allowing for dynamic changes in the effective exchange rate between action costs and the good values. We believe these properties are critical for maintaining adaptability in a changing environment. Throughout our evolutionary history, new opportunities and dangers constantly present themselves, making a fixed exchange rate between action costs and good value maladaptive.

The proposed computational framework can be conceived as analogous to classical value-comparison models in decision making, such as the drift diffusion model (DDM) [52] and the leaky competing accumulator (LCA) model [53], but for decisions that require continuous evaluation of in-flowing value information during ongoing actions. In the standard version of these models, choosing between two options is described by accumulator-to-threshold mechanisms. Sensory evidence associated with each alternative is accumulated, until the integrated evidence for one of them reaches a decision threshold. Despite the success of these frameworks to model a variety of decision tasks, they are difficult to extend beyond binary choices, require a pre-defined decision threshold and are mainly applied in perceptual decisions, in which decision precedes action. Unlike these models, the proposed computational theory can model decisions between multiple alternatives that either are presented simultaneously or sequentially, does not require any pre-defined decision threshold and can handle tasks in which subjects cannot wait to accumulate evidence before making a choice. The relative desirability integrates dynamically both sensory and motor evidence associated with a particular policy and reflects the degree to which this policy is best to follow at any given time and state with respect to the alternatives.

We tested our theory in a series of visuomotor decision tasks that involve reaching and saccadic movements and found that it captures many aspects of human and animal behavior observed in recent decision studies with multiple potential targets [11, 12, 21–23]. In line with these studies, the theory predicts the “delay-and-mix” behavior, when the competing goals have about the same good values and action costs and the “pre-selection” behavior, when one of the alternative goals is clearly the best option.

The present computational theory bears some similarities with the Hierarchical Reinforcement Learning (HRL) models used extensively in decision-making studies [54]. According to HRL theory, decision-making takes place at different level of abstractions, where the higher levels select the best current goal and the lower levels generate the optimal policy to implement the choice. Although, the HRL implements the dynamic aspects of decision-making by re-evaluating the alternative options and selecting the best one at a given time and state, there are two fundamental differences with the present theory. First, HRL always selects the best policy and typically pursues it until all the actions in the sequence have been performed. On the other hand, our computational theory generates a weighted average of the alternative policies and executes only part of it before re-evaluating the alternative option (i.e., see S5 Text about the “receding horizon control” theory). Second, the HRL uses a softmax transformation to evaluate the alternative options, whereas the proposed computational theory uses both the expected reward and the effort cost associated with each alternative. Additionally, other similar modular frameworks consisting of multiple control systems, such as the MOSAIC model [55] and the Q-decomposition framework [56], have been previously proposed to model tasks with multiple goals. However, these frameworks do not incorporate the idea of integrating both the good values and action costs into the action selection process. Hence, they fail to make predictions on how value information from disparate sources influences the motor competition and how this competition can lead to erroneous behavior.

### Decisions between conflicting options

We developed our model for cases where the competing options are similar. These are also cases where the relative effort and reward desirabilities are similar. For two options, it means the relative desirabilities would be far from zero or one. Here we consider extreme situations where one option requires much more effort or supplies much less reward. For extreme cases, the relative desirability calculation appears to break down and produces an “indeterminate” form for each alternative option. Here we explain why that happens, and how the indeterminacy is avoided by adding even a tiny amount of noise in implementing the calculation.

To illustrate the indeterminacy, consider selecting between an “extremely hard but very rewarding” and an “extremely easy but unrewarding” option. The hard option offers significantly higher reward than the easy option *reward*(*Hard*) > > *reward*(*Easy*), but it requires significantly higher effort to get it than the easy one *cost*(*Hard*) > > *cost*(*Easy*). According to the definition of the relative desirability, the reward-related component of the desirability will approach 1 for the hard option and 0 for the easy option, since *P*(*reward*(*Hard*) > *reward*(*Easy*)) = 1. On the other hand, the effort-related component of the desirability will be 0 for the hard option and 1 for the easy option, since *P*(*cost*(*Hard*) > *cost*(*Easy*)) = 1. The relative reliability multiplies these values and renormalizes, leading to the indeterminate form *rD*(*option*(1)) = 0*1/(0*1+1*0) = 0/0 and *rD*(*option*(2)) = 1*0/(0*1+1*0) = 0/0. In this case the model apparently fails to make a coherent choice. As long as the probability formula for reward and effort are continuous mappings, this indeterminacy will only be experienced in the limit that one option is infinitely harder to get (inaccessible) while the accessible option is comparably worthless.

However, the indeterminacy is an extreme example of an important class of problems where effort and reward values for the two options are in conflict with each other. Because there is a trade-off associated with *reward vs effort* neither option is clearly better than the other. While none of the decisions modeled here have extreme conflict, we nevertheless believe that the indeterminacy described above will never occur in a biological decision-making system due to the effects of even tiny amounts of noise on the relative desirability computation. If we assume that desirability values are the brain’s estimate of how “desirable” one option is with respect to alternatives in terms of expected outcome and effort cost, then it is reasonable to assume these estimates are not always precise. In other words, biological estimates of desirability should manifest stochastic errors, which we model by including noise in the estimates. In the S6 Text we show the effect of this noise is profound. For the extreme scenario in which *P*(*reward*(*Hard*) > *reward*(*Easy*)) = 1 and *P*(*cost*(*Hard*) > *cost*(*Easy*)) = 1, in the presence of noise the relative desirability of each option is 0.5. Thus, indeterminacy produces a lack of preference—since the “easy” option dominates the “hard” option in terms of effort, but the “hard” option is better than the “easy” option in terms of reward. In general, cases with extreme conflict will produce lack of preference, but these cases are also unstable—small changes in factors affecting the valuation such as the internal states of the subject (e.g., hunger level, fatigue level) can produce large shifts in preference. In the S6 Text, we further discuss the effects of noise in decisions with multiple options.

### Does the brain play dice?

One of the key assumptions in our study is that the brain continuously evaluates the relative desirability—i.e., the probability that a given policy will result in the highest pay-off with the least effort—in decisions with competing options. Although this idea is novel, experimental studies provide evidence that the brain maintains an explicit representation of “probability of choice” when selecting among competing options (for a review see [9]). For binary perceptual decisions, this probability describes the likelihood of one or another operant response, whereas for value-based decisions it describes the probability that selecting a particular option will result in the highest reward. Classic experimental studies reported a smooth relationship between stimulus parameters and the probability of choice suggesting that the brain translates value information to probabilities when making decisions [57, 58]. Additionally, neurophysiological recordings in non-human primates revealed activity related to the probability of choice in the lateral intraparietal area (LIP) both in “two-alternative force-choice eye movement decisions” and in “value-based oculomotor decisions”. In the first case, the animals performed the random-dot motion (RDM) direction discrimination task while neuronal activity was recorded from the LIP [59]. The activity of the LIP neurons reflects a general decision variable that is monotonically related to the logarithm of the likelihood ratio that the animals will select one direction of motion versus the other. In classic value-based decisions, the animals had to select between two targets presented simultaneously in both hemifields [15]. The activity of the LIP neurons is modulated by a number of decision-related variables including the expected reward and the outcome probability. These experimental findings have inspired previous computational theories to model perceptual- and value-based decisions [9]. According to these studies, when the brain is faced with competing alternatives, it implements a series of computations to transform sensory and value information into a probability of choice. The proposed idea of the relative desirability value can be conceived as an extension of these theories taking into account both the expected reward and the expected effort related to a choice.

### Action competition explains errors in behavior

One of the novelties of this theory is that it predicts not only successful decisions, but decisions that result in poor or incorrect actions. A typical example is the “global effect” paradigm that occurs frequently in short latency saccadic movements. When the goal elements are located in close proximity and subjects are free to choose between them, erroneous eye movements usually land at intermediate locations between the goals [24, 35]. Although the neural mechanisms underlying the global effect paradigm have not been understood fully yet, the prevailing view suggests that it occurs due to unresolved competition between the populations of neurons that encode the movements towards the two targets. Any target in the field is represented by a population of neurons that encodes the movement direction towards its location as a vector. The strength of the population is proportional to the saliency (e.g., size, luminance) and the expected pay-off of the target. When two similar targets are placed in close proximity, the populations corresponding to them will be combined to one mean population with the direction of the vector towards an intermediate location. If one of the targets is more salient or provide more reward than the other, the vector is biased to this target location. Since subjects have to perform saccadic movements to one of the targets, the competition between the two populations has to be resolved in time by inhibiting one of them. The time to suppress the neuronal activity that encodes one of the alternatives may be insufficient for short latency saccades resulting in averaging eye movements. Our findings are consistent with this theory. The strength of the neuronal population is consistent with relative desirability of the policy that drives the effector directed to the target. When the two equally rewarded targets are placed in close proximity, the two policies generate similar actions. Given that both targets are attached with the same goods-related values, the relative desirability of the two policies are about the same at different states, resulting in a strong competition. Because saccades are ballistic with little opportunity for correction during movement, the competition produces averaging saccades. On the other hand, placing the two targets in distance, the two saccadic policies generate dissimilar actions and consequently the competition is easier to be resolved in time.

Competition between policies in closely aligned goals can also explain errors in sequential decision tasks that involve serial order movements as described by Lashley [36]. The key idea in Lashley’s pioneer work (1951) is that the generation of serial order behavior involves the parallel activation of sequence of actions that are internally activated before each of the actions are executed. The main line of evidence of this hypothesis was the errors that occur frequently in serial order tasks, such as speech [37], typing [38], reaching [39] and copying of geometrical shapes [25]. For instance, a common error in typing and speaking is to swap or transpose nearby letters, even words. Lashley suggested that errors in sequential tasks would be most likely to occur when executing nearby elements within a sequence. Recent neurophysiological studies provide the neural basis of the Lashley’s hypothesis showing that the serial characteristics of a sequence of movements are represented in an orderly fashion in the prefrontal cortex, in time before the start of drawing [25, 40]. Training monkeys to copy geometrical shapes and recording the activity of individual neurons in the prefrontal cortex, the experimenters were able to identify populations of neurons that encode each of the segments [25]. The strength of the neuronal population corresponding to a segment predicted the serial position of the segment in the motor sequence. Interestingly, the temporal evolution of the strength of the segment representation during the execution of the trajectories for copying the shapes resembles the temporal evolution of the relative desirabilities of policies in our theory. This finding suggests that the strength of the neuronal population of a particular segment may encode the relative desirability (or components of the desirability) of copying that segment at a given time with respect to the alternatives. This hypothesis is also supported by error analysis in the serial order tasks, which showed that errors more frequently occurred when executing elements with nearly equal strength of representation. In a similar manner, our theory predicts that when two policies have about equal relative desirabilities over extended periods of the movement, the competition between them may lead to errors in behavior.

### A conceptual alternative in understanding the pathophysiology of the hemispatial neglect syndrome

Finally, our theory provides a conceptual alternative in understanding important aspects of neurological disorders that cause deficits in choice behavior, such as the spatial extinction syndrome. This syndrome is a subtle form of hemispatial neglect that occurs frequently after brain injury. It is characterized by the inability to respond to stimuli in the contralesional hemifield, but only when a simultaneous ipsilesional stimulus is also presented [60]. Recent studies reported contralesional bias that reminiscent the extinction syndrome, in oculomotor decision tasks after reversible pharmacological inactivation of the LIP [48] and the Pulvinar [61] in monkeys. According to our theory, this effect could be related to a deficit in value integration after inactivation, rather than simply sensory attention deficit.

### Conclusion

In sum, decisions require integrating both good values and action costs, which are often time and state dependent such that simple approaches pre-selection of goals or fixed weighted mixture of policies cannot account for the complexities of natural behavior. By focusing on a fundamental probabilistic computation, we provide a principled way to dynamically integrate these values that can merge work on decision making with motor control.

## Supporting Information

S1 FigEffects of noise in the relative desirability estimation.
**A**: We tested the effects of noise in the relative desirability estimation in a two-choice decision making task. The model was free to choose between the two targets (*g*_1_, *g*_2_) presented in different distances from the current hand position (*r*_1_, *r*_2_). Each of these targets offers reward that follows a Normal distribution $N\left(\mu_{1},\sigma_{1}^{2}\right),N\left(\mu_{2},\sigma_{2}^{2}\right)$. **B**: Heat map of the relative desirability value for selecting the target *g*_2_ as a function of the distance *r*_1_ and the expected reward *μ*_1_ of the alternative target *g*_1_. **C**: Relative desirability value for selecting the target *g*_2_ as a function of the distance *r*_1_ for different noise level *μ*_*ξ*_. **D**: Relative desirability value of selecting the target *g*_2_ in the “do-nothing vs. do-hard” decision (i.e., *r*_1_ = 0) as a function of the noise level *μ*_*ξ*_.(TIF)

S1 Text Stochastic optimal control theory for reaching movements. A detailed description of the stochastic optimal control theory used to model reaching movements to single targets.
(PDF)

S2 Text Stochastic optimal control theory for eye movements. A detailed description of the stochastic optimal control theory used to model eye movements to single targets.
(PDF)

S3 Text Inverse temperature parameter λ. A description of the inverse temperature parameter λ used in the transformation of the value-function to probability value in Eqs 3 and 4 (see the main manuscript).
(PDF)

S4 Text Updating the target probability based on history of trials. A description of the method used to update the target probability based on the trial history in rapid reaching movements.
(PDF)

S5 Text Receding horizon control. We implemented a receding horizon control technique to handle contingencies like changing the position of the targets, perturbations and effects of noise.
(PDF)

S6 Text Noise in the relative desirability estimation. We provide a detailed description of the effects of noise in the relative desirability estimation.
(PDF)
