# Supplementary figures and images for: Dynamic Integration of Value Information into a Common Probability Currency as a Theory for Flexible Decision Making
Source: PLoS Comput Biol. 2015 Sep 22;11(9):e1004402. doi: 10.1371/journal.pcbi.1004402 (PMC4578920; doi:10.1371/journal.pcbi.1004402)

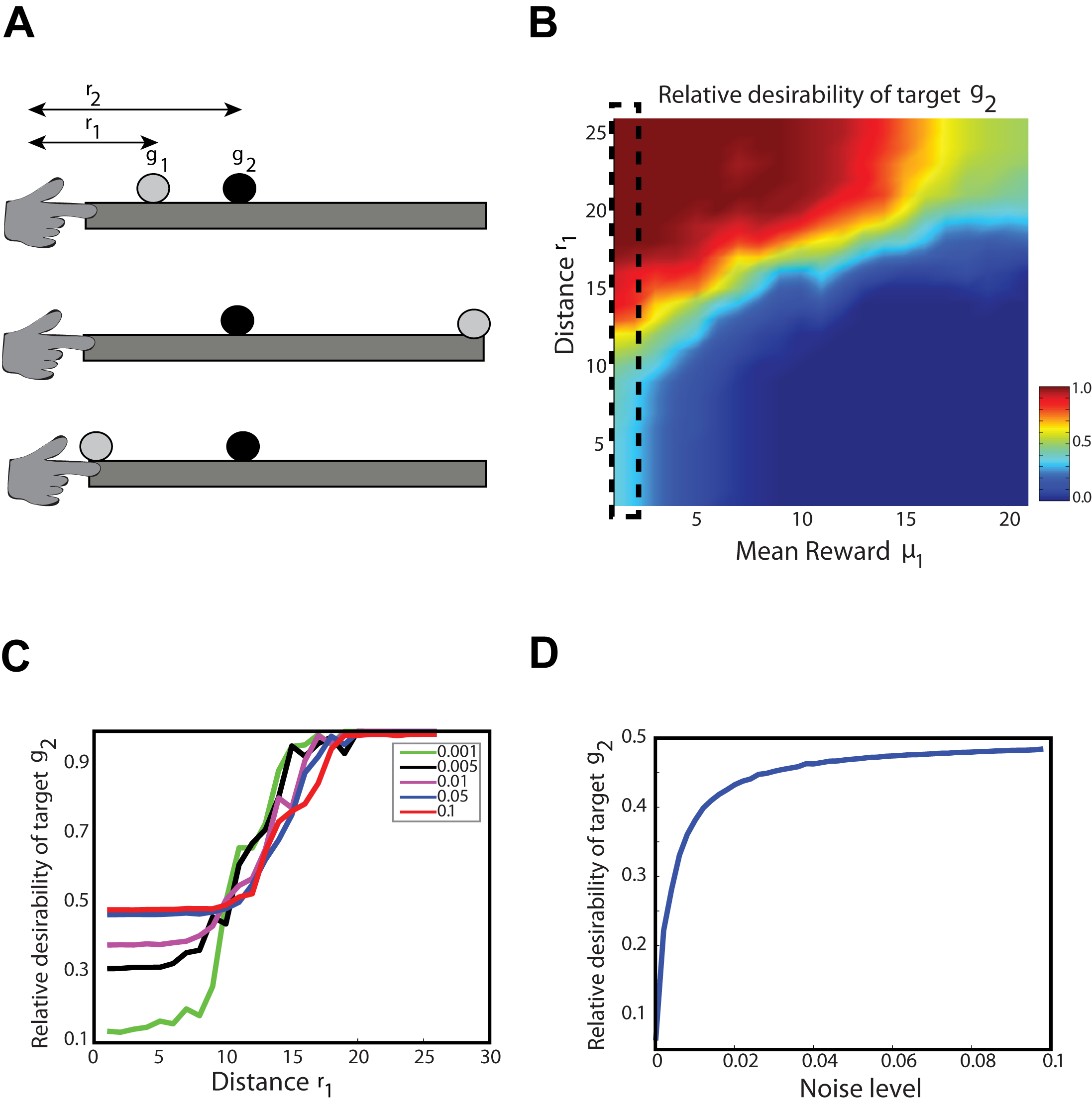

Supplement: S1 Fig — A: We tested the effects of noise in the relative desirability estimation in a two-choice decision making task. The model was free to choose between the two targets (g 1, g 2) presented in different distances from the current hand position (r 1, r 2). Each of these targets offers reward that follows a Normal distribution N(μ1,σ12),N(μ2,σ22). B: Heat map of the relative desirability value for selecting the target g 2 as a function of the distance r 1 and the expected reward μ 1 of the alternative target g 1. C: Relative desirability value for selecting the target g 2 as a function of the distance r 1 for different noise level μ ξ. D: Relative desirability value of selecting the target g 2 in the “do-nothing vs. do-hard” decision (i.e., r 1 = 0) as a function of the noise level μ ξ. (TIF) [file pcbi.1004402.s001.tif]
