# Supplementary material for: Dynamic Integration of Value Information into a Common Probability Currency as a Theory for Flexible Decision Making
Source: PLoS Comput Biol. 2015 Sep 22;11(9):e1004402. doi: 10.1371/journal.pcbi.1004402 (PMC4578920; doi:10.1371/journal.pcbi.1004402)
Supplement: S1 Text — A detailed description of the stochastic optimal control theory used to model reaching movements to single targets. (PDF) [file pcbi.1004402.s002.pdf]

S1 text

In S1 text, we provide details for the stochastic optimal control framework used to model reaching movements to single targets.

1 Hand modeling.

We modeled the dynamics of the hand as a “point of mass” (m = 1 Kg), in Cartesian coordinates, with 2-dimensional position $\mathbf{p}_t^h = [x_h(t), y_h(t)]^T$ and velocity $\dot{\mathbf{p}}_t^h = [\dot{x}_h(t), \dot{y}_h(t)]^T$ [1]. The combined actions of all muscles is represented by the force vector $\mathbf{f}_t = [f_x(t), f_y(t)]^T$. Neural control $\mathbf{u}_t$ is transformed into these forces $\mathbf{f}_t$ through second-order muscle-like low-pass filters with constants τ_1 and τ_2, and by adding control-dependent multiplicative noise [1].

$$\tau_1 \tau_2 \ddot{\mathbf{f}}_t + (\tau_1 + \tau_2)\dot{\mathbf{f}}_t + \mathbf{f}_t = \mathbf{u}_t \tag{1}$$

The second-order low pass filters can be written as a pair of coupled first-order filters with outputs $\mathbf{g}$ and $\mathbf{f}$, as:

$$\tau_1 \dot{\mathbf{g}}_t + \mathbf{g}_t = \mathbf{u}_t, \quad \tau_2 \dot{\mathbf{f}}_t + \mathbf{f}_t = \mathbf{g}_t \tag{2}$$

Assuming that the target position is on $\mathbf{p}^G = [x_G, y_G]^T$, the discrete-time state is described

through a 10th-dimensional vector $\mathbf{x}_t$, Eq. (3).

$$\mathbf{x}_t = [x_h(t), y_h(t), \dot{x}_h(t), \dot{y}_h(t), f_x(t), f_y(t), g_x(t), g_y(t), x_G, y_G]^T \tag{3}$$

The discrete-time dynamics of the hand are given by Eq. (4):

$$\begin{aligned}
\mathbf{p}^h_{t+\delta t} &= \mathbf{p}^h_t + \dot{\mathbf{p}}^h_t \delta t \\
\dot{\mathbf{p}}^h_{t+\delta t} &= \dot{\mathbf{p}}^h_t + \mathbf{f}_t \frac{\delta t}{m} \\
\mathbf{f}_{t+\delta t} &= \mathbf{f}_t \left(1 - \frac{\delta t}{\tau_2}\right) + \mathbf{g}_t \frac{\delta t}{\tau_2} \\
\mathbf{g}_{t+\delta t} &= \mathbf{g}_t \left(1 - \frac{\delta t}{\tau_1}\right) + \mathbf{u}_t \left(1 + \sigma_c \epsilon_t\right) \frac{\delta t}{\tau_1}
\end{aligned} \tag{4}$$

where δt = 0.01s is the sampling period of discretization. The product term $\sigma_c \epsilon_t \mathbf{u}_t$ describes the multiplicative noise added to the control signal $\mathbf{u}_t$, with $\sigma_c = 1$, which is a unitless variable defined as the noise magnitude related to the control signal magnitude [1].

2 Reaching to a single target.

Within the stochastic optimal control framework, control policies originate as the solution to an optimization problem. The basic idea is to find a sequence of motor commands that acquire as

much reward as possible, while spending as little effort as possible. For reaching, given the arm's kinematics and the sensory and motor noise in estimating and controlling state of the hand, the stochastic optimal control finds a policy $\mathbf{u}_t^* = \pi^*(\mathbf{x}_t)$, for time instances $t = [t_1, \ldots, t_{end}]$ that optimizes the cost function J described in Eq. (5), for each state of the hand and the environment $\mathbf{x}_t$.

$$J(\mathbf{x}_t, \pi(\mathbf{x}_t)) = \|\mathbf{p}^h_{t_{end}} - \mathbf{p}^G\|^2 + \|\dot{\mathbf{p}}^h_{t_{end}}\|^2 + \|\mathbf{f}_{t_{end}}\|^2 + \sum_{t=1}^{t_{end}-1} \pi(\mathbf{x}_t)^T R \pi(\mathbf{x}_t) \tag{5}$$

The first term is the accuracy cost and penalizes policies that drive the end-point of the reaching trajectory away from the target position $\mathbf{p}^G$. The second and the third terms specify that the reaching movement should stop at time t_{end} and the last term is the motor command cost that penalizes the effort required to reach the target. The matrix $R = \frac{1}{t_{end}} \begin{bmatrix} r_x & 0 \\ 0 & r_y \end{bmatrix}$ is the control-dependent cost of the hand motion in the x and y dimension $(r_x = r_y = 0.001)$.

We can write Eq. (5) in the general form of optimal control cost function as follows:

$$J = \left(\mathbf{x}_{t_{end}} - S\mathbf{p}^G\right)^T Q_{t_{end}} \left(\mathbf{x}_{t_{end}} - S\mathbf{p}^G\right) + \sum_{t=1}^{t_{end}-1} \pi(\mathbf{x}_t)^T R \pi(\mathbf{x}_t) \tag{6}$$

where S is a matrix that picks out the hand and target positions from the state vector. The time-varying matrix Q_t describes the state-dependent cost and is the zero matrix for any time

$t < t_{end}$ and is equal to the Hessian matrix of the cost function evaluated at the end of the movement t_{end}.

To minimize the cost function in Eq. (6), a model of the system dynamics and sensory feedback must be incorporated. Abundant evidence suggests the sensory system uses internal forward models that predict the next state at time $t+1$, $\hat{\mathbf{x}}_{t+1|t}$, based on the sensory feedback $\mathbf{y}_t$, the current state estimate $\hat{\mathbf{x}}_t$ and the control commands $\mathbf{u}_t$ [2], which helps overcome control instabilities due to noisy sensors and temporal delays. Following, we modeled the hand and the state space using linear dynamics and measurement as a discrete linear system, Eq. (7), considering the motor commands are corrupted by multiplicative noise, normally distributed with zero mean and standard deviation proportional to the magnitude of the control commands and the state variables [1].

$$\begin{aligned} \mathbf{x}_{t+1} &= A\mathbf{x}_t + B\mathbf{u}_t + \xi_t + C(\mathbf{u}_t)\epsilon_t \\ \mathbf{y}_t &= H\mathbf{x}_t + \omega_t \end{aligned} \tag{7}$$

where A, B and H are the actual system dynamics and observation matrices, respectively.

$$A = \begin{bmatrix} 1 & 0 & \delta t & 0 & 0 & 0 & 0 & 0 & 0 & 0 \\ 0 & 1 & 0 & \delta t & 0 & 0 & 0 & 0 & 0 & 0 \\ 0 & 0 & 1 & 0 & \frac{\delta t}{m} & 0 & 0 & 0 & 0 & 0 \\ 0 & 0 & 0 & 1 & 0 & \frac{\delta t}{m} & 0 & 0 & 0 & 0 \\ 0 & 0 & 0 & 0 & 1-\frac{\delta t}{\tau_2} & 0 & \frac{\delta t}{\tau_2} & 0 & 0 & 0 \\ 0 & 0 & 0 & 0 & 0 & 1-\frac{\delta t}{\tau_2} & 0 & \frac{\delta t}{\tau_2} & 0 & 0 \\ 0 & 0 & 0 & 0 & 0 & 0 & 1-\frac{\delta t}{\tau_1} & 0 & 0 & 0 \\ 0 & 0 & 0 & 0 & 0 & 0 & 0 & 1-\frac{\delta t}{\tau_1} & 0 & 0 \\ 0 & 0 & 0 & 0 & 0 & 0 & 0 & 0 & 1 & 0 \\ 0 & 0 & 0 & 0 & 0 & 0 & 0 & 0 & 0 & 1 \end{bmatrix}$$

$$B = \begin{bmatrix} \mathbf{0}_{6\times 2} \\ \begin{matrix} \frac{\delta t}{\tau_1} & 0 \\ 0 & \frac{\delta t}{\tau_1} \end{matrix} \\ \mathbf{0}_{2\times 2} \end{bmatrix}, H = \begin{bmatrix} \mathbf{I}_{6\times 6} \mid \mathbf{0}_{6\times 4} \end{bmatrix} \tag{8}$$

The noise variables ξ_t, ω_t and ϵ_t are normally distributed variables with zero mean and covariance $\Omega^\xi \geq 0$, $\Omega^\omega \geq 0$ and $\Omega^\epsilon = I$, respectively. $C(\mathbf{u}_t)$ is a scaling matrix for control-dependent

system noise, such as $C(\mathbf{u})\epsilon = \sum_i C_i \mathbf{u} \epsilon^i$, where ϵ^i is the ith component of the random variable ϵ.

Given the belief about the state at time t and the goal of the task, the stochastic optimal controller generates the optimal policy - the best sequence of actions that we can perform to reach the goal - $\pi^* (\mathbf{x}_t) = \mathbf{u}_t^*$ that minimizes the expected cost function in Eq. (5). This form of optimal control is a modified Linear Quadratic Gaussian (LQG) regulator, since the dynamics of the system are linear, the expected cost function is Quadratic and the noise is Gaussian, but with signal-dependent noise [1].

Incorporating the optimal policy into the system model generates a feedback controller that uses its forward model to generate predictions $\hat{\mathbf{y}}_t$ from knowledge of controls, dynamics and sensory measurements, and it combines predictions with sensory feedback $\mathbf{y}_t$ via Eq. (9) to update the belief about the state in time $t + 1$.

$$\hat{\mathbf{x}}_{t+1|t+1} = (A - BL_t)\hat{\mathbf{x}}_{t+1|t} + K_t \left(\mathbf{y}_t - H\hat{\mathbf{x}}_{t+1|t} \right) \tag{9}$$

where K_t describes the Kalman gain at the given time t.

References

1. Todorov E (2005) Stochastic optimal control and estimation methods adapted to the noise characteristics of the sensorimotor system. Neural Comput. 17: 1084-1108.

2. Diedrichsen J, Shadmehr R, Ivry R (2010) The coordination of movement. Optimal feedback control and beyond. Trends Cogn Sci. 14: 31-39.
