# Supplementary material for: Dynamic Integration of Value Information into a Common Probability Currency as a Theory for Flexible Decision Making
Source: PLoS Comput Biol. 2015 Sep 22;11(9):e1004402. doi: 10.1371/journal.pcbi.1004402 (PMC4578920; doi:10.1371/journal.pcbi.1004402)
Supplement: S2 Text — A detailed description of the stochastic optimal control theory used to model eye movements to single targets. (PDF) [file pcbi.1004402.s003.pdf]

# S2 text

In S2 text, we provide details for the stochastic optimal control framework used to model saccade movements to single targets.

**Eye modeling.** We modeled the globe and the surrounding tissues using an elastic element with stiffness $k$ that pull the eye away from the equilibrium point ($x = 0$), and a viscous element $\mu$ that resists in this motion (for more details about this eye model see [1] ). The one-dimensional dynamics of the eye is given by Eq. (1).

$$m\ddot{x} = -kx - \mu\dot{x} + u \tag{1}$$

where $m$ is the inertia of the eye and $u$ are the motor commands.

We considered a simple model that translates motor commands into forces, such as: $\alpha_1 \dot{f} + \alpha_2 f = u$, where $f$ describes the instantaneous force generated by the extra-ocular muscles to move the eye.

Assuming that the target position is on $\mathbf{p}^G = [x_G, y_G]^T$, the discrete-time state is described through a $8^{th}$-dimensional vector $\mathbf{x}_t$, Eq. (2).

$$\mathbf{x}_t = [x_e(t), \dot{x}_e(t), y_h(t), \dot{y}_e(t), f_x, f_y, x_G, y_G]^T \tag{2}$$

The two-dimensional discrete-time dynamics of the eye are given by Eq. (3):

$$\begin{aligned}
\mathbf{p}^e_{t+\delta t} &= \mathbf{p}^e_t + \dot{\mathbf{p}}^e_t \delta t \\
\dot{\mathbf{p}}^e_{t+\delta t} &= -\delta t \frac{k}{m} \mathbf{p}^e_t + \left(1 - \delta t \frac{\mu}{m}\right) \dot{\mathbf{p}}^e_t + \frac{\delta t}{m} \mathbf{f}_t \\
\mathbf{f}_{t+\delta t} &= \left(1 - \delta t \frac{\alpha_2}{\alpha_1}\right) f_t + \mathbf{u}_t \left(1 + \sigma_c \epsilon_t\right) \frac{\delta t}{\alpha_1}
\end{aligned} \tag{3}$$

where $\mathbf{p}^e_t = [x_e(t), y_e(t)]^T$ and $\dot{\mathbf{p}}^e_t = [\dot{x}_e(t), \dot{y}_e(t)]^T$ is the 2-dimensional position and velocity of the eye, respectively. Similarly, with the hand model, the product term $\sigma_c \epsilon_t \mathbf{u}_t$ describes the multiplicative noise added to the control signal $\mathbf{u}_t$, with $\sigma_c = 1$ and $\epsilon_t$ a vector of zero-mean random variables with covariance $\Omega^e = \mathbf{I}$. Additionally, $\delta t = 0.001$s is the sampling period of discretization.

**Saccade to a single target.** Saccadic eye movements are so brief in time that the sensory feedback information seems to have no role on the control of these movements. However, this does not imply that saccades are open-loop movements. Instead, recent findings suggest that motor commands that generate saccades are not pre-programmed and benefit from a forward internal model that monitors the motor commands and predicts the consequence of them [2].

We modeled the saccadic eye movements using a similar optimal control framework with the reaching movements, with the difference that the sensory feedback information does not affect the control of the saccadic eye movements. In this framework, the problem is to generate these motor commands that bring the eye to a certain location in a given time interval, while minimizing the total effort. This cost function is the same to the one that used on modeling reaching movements to single targets (see Eq. (6) S1 text).

The dynamics of the eye can be transformed into the form of Eq. (7) in S1 text, with the following matrices:

$$A = \begin{bmatrix} 1 & \delta t & 0 & 0 & 0 & 0 & 0 & 0 \\ -\frac{k\delta t}{m} & 1-\frac{\mu\delta t}{m} & 0 & 0 & \frac{1}{m}\delta t & 0 & 0 & 0 \\ 0 & 0 & 1 & \delta t & 0 & 0 & 0 & 0 \\ 0 & 0 & -\frac{k\delta t}{m} & 1-\frac{\mu\delta t}{m} & 0 & \frac{1}{m\delta t} & 0 & 0 \\ 0 & 0 & 0 & 0 & 1-\frac{\alpha_1\delta t}{\alpha_2} & 0 & 0 & 0 \\ 0 & 0 & 0 & 0 & 0 & 1-\frac{\alpha_1\delta t}{\alpha_2} & 0 & 0 \\ 0 & 0 & 0 & 0 & 0 & 0 & 1 & 0 \\ 0 & 0 & 0 & 0 & 0 & 0 & 0 & 1 \end{bmatrix}$$

$$B = \begin{bmatrix} 0_{4\times 2} \\ \begin{matrix} \frac{\delta t}{\alpha_1} & 0 \\ 0 & \frac{\delta t}{\alpha_1} \end{matrix} \\ 0_{2\times 2} \end{bmatrix} \quad (4)$$

For a monkey eye, we used time constants $\tau_1 = 0.260$ s and $\tau_2 = 0.012$ s as proposed by Keller [3] and Robinson et al. [4]. These time constants are related with the constants of the system dynamic matrices $A$ and $B$ as follows: $\mu = \tau_1 + \tau_2$, $m = \tau_1 \tau_2$. Based on these studies, we set $k = 1$, $\alpha_1 = 0.004$ and $\alpha_2 = 1$.

## References


1. Haith A, Reppert R, Shadmehr R (2012) Evidence for hyperbolic temporal discounting of reward in control of movements. J Neurosci. 32: 11727–11736.

2. Ethier V, Zee D, Shadmehr R (2010) Changes in control of saccades during gain adaptation. J Neurosci. 28: 13929-13937.

3. Keller E (1973) Accommodative vergence in the alert monkey. Motor unit analysis. Vision Res. 13: 1565-1575.

4. Robinson D, Gordon J, Gordon S (1986) A model of the smooth pursuit eye movement system. Biol Cybern. 55: 43–57.
