# Supplementary material for: Dynamic Integration of Value Information into a Common Probability Currency as a Theory for Flexible Decision Making
Source: PLoS Comput Biol. 2015 Sep 22;11(9):e1004402. doi: 10.1371/journal.pcbi.1004402 (PMC4578920; doi:10.1371/journal.pcbi.1004402)
Supplement: S3 Text — A description of the inverse temperature parameter λ used in the transformation of the value-function to probability value in Eqs 3 and 4 (see the main manuscript). (PDF) [file pcbi.1004402.s004.pdf]

S3 text

One of the key computations in the present framework is the transformation of the value function $V_{\pi_j}(\mathbf{x}_t)$ to probability value using a soft-max type equation (see Eq. 3 and Eq. 4 in the main manuscript). It characterizes the probability of spending at least effort $V_{\pi_j}(\mathbf{x}_t)$ starting from the current state $\mathbf{x}_t$ at time t and adopting the policy $\pi_j(\mathbf{x}_t)$ to move to the target j. The free parameter in this transformation is the inverse temperature λ. The values of λ were chosen empirically, but the qualitatively predictions presented in the manuscript are relative robust to the chosen values of λ. Note that the same value of λ was used in every simulated experiment. Particularly, we set $\lambda = 0.1$ for the rapid-reaching experiments, $\lambda = 0.01$ for the eye-movement experiments and $\lambda = 0.5$ for the sequential reaching (i.e., copy geometrical shapes) experiment.
