# Supplementary material for: Dynamic Integration of Value Information into a Common Probability Currency as a Theory for Flexible Decision Making
Source: PLoS Comput Biol. 2015 Sep 22;11(9):e1004402. doi: 10.1371/journal.pcbi.1004402 (PMC4578920; doi:10.1371/journal.pcbi.1004402)
Supplement: S4 Text — A description of the method used to update the target probability based on the trial history in rapid reaching movements. (PDF) [file pcbi.1004402.s005.pdf]

S4 text

We incorporate the history of trials in the computational framework by varying the target probability $p(target = j)$ with the frequency that the target j was cued for action on the past trials. We update the value of the probability on each trial using a simple reinforcement learning algorithm. This algorithm estimates the target probability $p_{k+1}(target = j)$ at trial $k + 1$ given the recent trial history, Eq. (1).

$$p_{k+1}(target = j) = p_k(target = j) + \alpha(C - p_k(target = j)) \tag{1}$$

where k is the current trial. We set $C = 1$, if the target j was cued for action on the current trial, otherwise $C = 0$. Also, α is the learning rate that was set to 0.4 on the simulations.
