# Supplementary material for: Dynamic Integration of Value Information into a Common Probability Currency as a Theory for Flexible Decision Making
Source: PLoS Comput Biol. 2015 Sep 22;11(9):e1004402. doi: 10.1371/journal.pcbi.1004402 (PMC4578920; doi:10.1371/journal.pcbi.1004402)
Supplement: S5 Text — We implemented a receding horizon control technique to handle contingencies like changing the position of the targets, perturbations and effects of noise. (PDF) [file pcbi.1004402.s006.pdf]

S5 text

In modeling visually-guided movements in natural environments, the controller should be able to handle contingencies like changing the position of the targets, perturbations and effects of noise and others. These contingencies affect the movement duration - it increases for correcting movements - and therefore a fixed-horizon formulation of the optimal control model does not capture the temporal variability of the movement [1]. To overcome these problems, we implement a receding-horizon control strategy [2,3]. Receding horizon control recomputes optimal fixed time policies iteratively from the current state $\mathbf{x}_t$ to the goal state $\mathbf{x}_{t_{end}}$, executing only the initial portion of this policy for a short period of time k and then it recomputes a new optimal policy from the state $\mathbf{x}_{t+k}$ until the time $t + k + t_{end}$ and so on, until the reach is completed. In our problem, we computed the individual policies for reaching the targets from the current state to the goal, but we derived the averaging policy using only the first $k = 10$ time steps of these policies. Then, we recomputed the new policies from the current state to the end and so on, until the hand reaches one of the targets.

References

1. Liu D, Todorov E (2007) Evidence for the flexible sensorimotor strategies predicted by optimal feedback control. J Neurosci. 27: 9354–9368.

2. Bemporad A, Morari M (1999) Robust model predictive control: A survey. Robustness in Identification and Control 245: 207–226.

3. Goodwin G, Seron M, de Dona J (2005) Constrained Control and Estimation: An Optimisation Approach. Springer, London, UK.
