# Supplementary material for: Dynamic Integration of Value Information into a Common Probability Currency as a Theory for Flexible Decision Making
Source: PLoS Comput Biol. 2015 Sep 22;11(9):e1004402. doi: 10.1371/journal.pcbi.1004402 (PMC4578920; doi:10.1371/journal.pcbi.1004402)
Supplement: S6 Text — We provide a detailed description of the effects of noise in the relative desirability estimation. (PDF) [file pcbi.1004402.s007.pdf]

S6 text

In the main manuscript, we discussed how indeterminacy can result from cases where the reward and effort desirabilities are in extreme conflict. These cases involve options of very high value that are hard to get pitted against easy to get options that are comparably worthless. Here we look at the effect of small errors in the desirability computations on the relative desirability as a function of the conflict between options.

To simulate errors in desirability computations, we added Gaussian noise to the effort and reward desirabilities, truncating to ensure the quantities remained between zero and one. To illustrate the effects of these errors, consider a family of tasks that involve reaching between two targets g_1 and g_2, located in distance r_1 and r_2 from the current hand position, respectively, and offer reward that follows a Normal distribution $N(\mu_1, \sigma^2)$ and $N(\mu_2, \sigma^2)$, respectively, Fig S1 A top panel. The relative desirability for reaching the target g_1 and g_2 from the current hand position at the state $\mathbf{x}_t$ is given as:

$$\begin{aligned} rD\left(\pi_{g_1}\left(\mathbf{x}_t\right)\right) &= P\left(cost(g_1) < cost(g_2)\right) P\left(reward(g_1) > reward(g_2)\right) + \xi \\ rD\left(\pi_{g_2}\left(\mathbf{x}_t\right)\right) &= P\left(cost(g_2) < cost(g_1)\right) P\left(reward(g_2) > reward(g_1)\right) + \xi \end{aligned} \tag{1}$$

where π_{g_i} is the optimal policy to get to the target g_i, and ξ is the simulated error in the relative desirability estimation that sampled from a Normal distribution $N(\mu_\xi, \sigma_\xi^2)$.

We ensure the rD values are between zero and one and normalized the relative desirability

values of the two options so that they all sum to 1:

$$rD^{Norm}\left(\pi_{g_i}\left(\mathbf{x}_t\right)\right) = \frac{rD\left(\pi_{g_i}\left(\mathbf{x}_t\right)\right)}{\sum_{i=1}^{2} rD\left(\pi_{g_i}\left(\mathbf{x}_t\right)\right)}, i = 1, 2 \quad (2)$$

For notational simplicity, we omit the $Norm$ sign from the relative desirability, and from now on $rD\left(\pi_{g_i}\left(\mathbf{x}_t\right)\right)$ will indicate the normalized relative desirability of the target g_i at the current state $\mathbf{x}_t$.

Let's assume that the target g_2 is located at a distance $r_2 = 15$ steps from the current hand position and offers reward that follows a Normal distribution $N(8, 0.05)$. Additionally, the distribution of the noise ξ is Normal with mean and variance: $N(0.01, 0.0005)$. Fig S1 B depicts the heat map of the relative desirability values for selecting the target g_2 as a function of the distance r_1 and the expected reward μ_1 of the alternative target g_1. In other words, it describes how desirable is to select the target g_2 given the current location and the current expected reward of the target g_1. Notice that the upper right and bottom left corners of the heat map correspond to "conflicting decisions", in which the two options do not dominate each other, since one of them is significantly better in terms of the expected outcome and the alternative is better in terms of the effort cost. In this case, the two options have about the same relative desirability value, since they have a trade-off associated with reward vs. effort.

An interesting question is how the level of noise ξ affects the choice preference in this decision problem. To address this question, we fixed the expected reward of target g_1 to $\mu_1 = 1$ and varied the distance r_1 (i.e., we "sliced" through the Fig S1 B by fixing the expected reward of the

target g_1, see the discontinuous rectangle). The reward-related component of the relative desirability for selecting the target g_1 is $P(reward(g_1) > reward(g_2)) = \epsilon$, where $\epsilon << 1$ (but not zero), since the expected outcome for selecting the target g_1 is significantly lower than the expected outcome for selecting the target g_2. Additionally, let's assume that the effort-related component of the relative desirability for selecting the target g_2 is $P(cost(g_1) > cost(g_2)) = k$, where k can be any number between 0 and 1.

The reward- and effort-related components of the relative desirability values for these two options are given bellow:

$$\begin{aligned}P(reward(g_1) > reward(g_2)) &= \epsilon \\ P(reward(g_1) < reward(g_2)) &= 1-\epsilon \\ P(cost(g_1) > cost(g_2)) &= k \\ P(cost(g_1) < cost(g_2)) &= 1-k \end{aligned} \tag{3}$$

The relative desirability value for choosing the target g_2 is given as:

$$rD\left(\pi_{g_2}\left(x_t\right)\right) = \frac{\left(1-\epsilon\right)k+\xi}{\left(1-\epsilon\right)k+\xi+\epsilon(1-k)+\xi} = \frac{\left(1-\epsilon\right)k+\xi}{\left(1-2\epsilon\right)k+2\xi+\epsilon} \tag{4}$$

When the target g_1 is located further away than the target g_2 from the current hand position (see Fig S1 A middle panel), the model has to decide between one option that provides high reward and requires low effort and an alternative that provides low reward and requires high effort. In this case, g_2 is clearly the best option, since it requires lower effort and provides higher reward than the

alternative option (i.e., upper left region of the heat map in Fig S1 B). Hence, the effort component of the desirability for getting the g_1 tends to 1 (i.e., $k \to 1$), and the relative desirability for selecting the target g_2 is given from Eq. (4), as:

$$rD\left(\pi_{g_2}\left(x_t\right)\right) = \frac{1-\epsilon+\xi}{1-\epsilon+\xi+\xi} = \frac{1}{1+\frac{\xi}{1-\epsilon+\xi}} \tag{5}$$

Since ϵ and ξ are small numbers, the ratio $\frac{\xi}{1-\epsilon+\xi}$ is also a small number and hence, the noise has minimal effect on the relative desirability and so on the choice preference.

An interesting case occurs when the target g_1 is very close to the hand (i.e., bottom left region of the heat map in Fig S1 B). In this scenario the model has to decide between two conflicting options; An "easy" target that offers low reward and a "hard" target that provides high reward. This is similar to a scenario in which you are asked to decide between an option of doing nothing with a chance of receiving a low reward or you can choose an alternative goal with a chance of getting a significantly higher reward (see Fig S1 A bottom panel). In this case, the effort for reaching to the target g_2 is significantly higher than the effort for staying with target g_1 and therefore $k \to 0$. Hence, the relative desirability for choosing the target g_2 (i.e., the hard option) becomes:

$$rD\left(\pi_{g_2}\left(x_t\right)\right) = \frac{\xi}{\epsilon+2\xi} = \frac{1}{\frac{\epsilon}{\xi}+2} \tag{6}$$

Notice that in this case the relative desirability for the target g_2 depends on how large the noise level is with respect to ϵ. When the noise level is significantly lower than ϵ , i.e., $\xi << \epsilon$, the relative desirability of selecting the target g_2 tends to 0, which means that the model chooses to *exploit* the current choice (i.e., do nothing and receive a low amount of reward). On the other

hand, when the noise level is significantly higher than ϵ, i.e., $\xi >> \epsilon$, the relative desirability for selecting the hard option tends to 0.5, which means that the model will $explore$ the environment selecting between the "do nothing" and "do hard" options with about the same probability. These results suggest that the noise level has a significant impact on the choice preference when selecting between conflicting options. In the extreme scenario that we discussed in the main manuscript (see Discussion subsection "Decisions between conflicting options"), where $\epsilon = 0$, both of the alternative options have the same relative desirability values (i.e., $rD\left(\pi_{g_1}\left(x_t\right)\right) = rD\left(\pi_{g_2}\left(x_t\right)\right) = 0.5$), and hence the model selects either of these two options with equal probability.

The effect of noise level in the choice preference is illustrated in Fig S1 C which shows the relative desirability for selecting the target g_2 as a function of the distance r_1 for different noise level μ_ξ. As we discussed before, the noise level affects mainly the relative desirability values for conflicting choices (i.e., target g_1 is closer to the hand than the alternative g_2 target). On the other hand, the noise level has almost no effect on the desirability values when one option clearly outperforms the other. Finally, Fig S1 D depicts the relative desirability of selecting the target g_2 in the "do-nothing vs. do-hard" decision (i.e., $r_1 = 0$) as a function of the noise level. Consistent with what we discussed before, the model tends to select the do-nothing option for low level noise, whereas the possibility to explore the do-hard option increases with the noise level.

This discussion suggests that our framework can make interesting predictions even in cases of extreme conflict. In particular, cases of conflict are highly sensitive to small changes in the estimates of reward and effort, and thus we expect to see the biggest "changes of mind" due to

learning in these cases. As an example from everyday life, consider the decision for booking a flight. Usually, you have to decide between buying a cheap ticket with multiple layovers or an expensive non-stop ticket. If this is the first time that you are flying, it is likely that you will select a layover flight to save some money, because as a first time flyer you have a poor estimate of the desirability of non-stop flights with respect to flights with layovers. On the other hand, if you are a frequent flyer, you know how exhausting flight with long and many layovers can be, and therefore you have a more accurate estimate of the relative desirability of these two options. Hence, in this situation, it is likely that you will select a direct flight, although it is more expensive. On the other hand, if you are lucky enough to choose between a cheap non-stop flight and an expensive flight with many layovers, it is almost certain that you will select the first one irrespective of the noise in estimating the relative desirabilities of these two options. Overall, our model provides some interesting predictions about the choice preference in decisions between conflicting options. Further investigation is needed to test whether these predictions are consistent with human and animal behavior in high conflict tasks with learning.
